# Pathogen‐Centric Activation of an Azoreductase‐Responsive Antibody‐Antibiotic Conjugate for the Targeted Eradication of MRSA

**DOI:** 10.1002/advs.75541

**Published:** 2026-05-11

**Authors:** Qi Cheng, Lianqi Liu, Chenghua Liu, Fei Xie, Jingwen Dong, Xiaoyu Qin, Shunxiang Huang, Xian Li, Xingyuan Kou, Hongbin Deng, Jiannan Feng, Wu Zhong, Dian Xiao, Xinbo Zhou

**Affiliations:** ^1^ School of Pharmacy Qingdao University Qingdao China; ^2^ Academy of Military Medical Sciences Beijing China; ^3^ National Engineering Research Center for the Emergency Drug Beijing China; ^4^ School of Pharmaceutical Engineering Shenyang Pharmaceutical University Shenyang China; ^5^ Institute of Medicinal Biotechnology, Chinese Academy of Medical Sciences and Peking Union Medical College Beijing China

**Keywords:** acute infection, antibody‐antibiotic conjugates, azoreductase, bacterial enzyme, MRSA, peritonitis, septicemia

## Abstract

Antibody‐antibiotic conjugates (AACs) provide a transformative platform for eradicating intracellular methicillin‐resistant *Staphylococcus aureus* (MRSA). However, current AACs largely follow antibody‐drug conjugates (ADCs) design principles and rely on host‐lysosomal proteases for activation, rendering them ineffective against extracellular (planktonic) bacteria. Furthermore, we demonstrate that high bacterial burden induces host‐lysosomal dysfunction, fundamentally compromising the efficacy of host‐dependent AACs against intracellular reservoirs. To address these limitations, we developed a pathogen‐centric AAC (AZO‐AAC) featuring an azobenzene‐based linker that is triggered by bacterial‐secreted azoreductase. This strategy shifts the activation mechanism from host‐cell machinery to the pathogen itself, enabling antibiotic release independent of host‐cell integrity. Surprisingly, our designed AZO‐AAC achieves nanomolar‐scale eradication of both planktonic and intracellular MRSA, reaching levels below the limit of detection in scenarios where traditional AACs fail. In murine models of peritonitis and septicemia, a single dose (60 mg/kg) of AZO‐AAC resulted in a six‐log reduction in bacterial burden and the preservation of normal tissue architecture. By decoupling activation from host‐cell status, this pathogen‐responsive platform provides a robust strategy for the targeted elimination of complex, multi‐niche MRSA infections.

## Graphical abstract

A pathogen‐centric antibody‐antibiotic conjugate (AZO‐AAC) releases antibiotics via bacterial azoreductase, independent of host‐lysosomal function. This mechanism achieves nanomolar eradication of both planktonic and intracellular methicillin‐resistant *Staphylococcus aureus* (MRSA). A single dose reduces bacterial burden by six logs in murine peritonitis and septicemia models, overcoming host‐dependent AAC limitations.

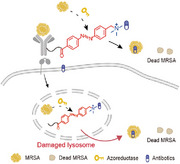

## 1 Introduction

The global health crisis caused by *Staphylococcus aureus* (*S. aureus*), particularly the multidrug‐resistant form methicillin‐resistant *S. aureus* (MRSA), is escalating due to its ability to render all *β*‐lactam antibiotics ineffective [1]. The persistence of MRSA is associated with its complex adaptive landscape, primarily characterized by biofilm formation [2, 3] and the establishment of stable intracellular reservoirs within professional phagocytes such as macrophages [4]. These “sanctuaries” can shield MRSA from systemic antibiotic exposure and host immune surveillance, often contributing to treatment failure and clinical relapse [5, 6]. Even glycopeptides, considered the “last line of defense” (e.g., vancomycin) [7], fail to achieve complete eradication due to their poor cell membrane permeability and limited intracellular activity.

To improve intracellular antibacterial delivery, antibody–antibiotic conjugates (AACs) have emerged as a transformative therapeutic modality [8, 9]. Pioneered by Lehar and colleagues, this strategy leverages antibody‐mediated endocytosis to deliver potent antimicrobial agents directly to intracellular niches [10]. The current design paradigm depends on host‐lysosomal proteases—particularly cathepsin B (CTSB) [9, 11], which cleaves peptide linkers such as Val‐Cit and releases the antibiotic payload. Although this host‐centric strategy has demonstrated efficacy against persistent, drug‐resistant bacteria in experimental models, its translation to complex clinical infections faces significant challenges [12, 13].

The fundamental Achilles’ heel of current AACs is their dependence on intact host‐lysosomal function. Critically, several works have found that severe MRSA infections often induce lysosomal membrane permeabilization (LMP) [14, 15] and phagocytic dysfunction [16], a process potentially driven by MRSA‐secreted pore‐forming toxins and other virulence factors [17]. In these compromised micro‐environments, host CTSB‐mediated activation becomes highly inefficient [18, 19], leading to sub‐therapeutic drug release and the consequent failure of clinical candidates such as DSTA4637S [20, 21]. These observations underscore the pressing need for a pathogen‐centric AAC platform capable of decoupling drug activation from the health status of the host‐cell.

A key direction for ADCs is to leverage tumor microenvironment‐specific conditions, such as high glutathione levels or extracellular proteases, to achieve linker cleavage and payload release outside target cells [22]. However, AACs that leverage the infection microenvironment for payload release have rarely been reported. Tvilum et al. designed a disulfide‐linked AAC that undergoes thiol–disulfide reshuffling with exofacial thiols on the bacterial surface for targeted payload release without internalization, showing superior antimicrobial activity in vitro and in vivo in an osteomyelitis model [23]. On the other hand, in fact, bacteria themselves also secrete a variety of enzymes, such as *β*‐lactamases, nitroreductases, azoreductases, and quinone reductases [24, 25].

**FIGURE 1:**
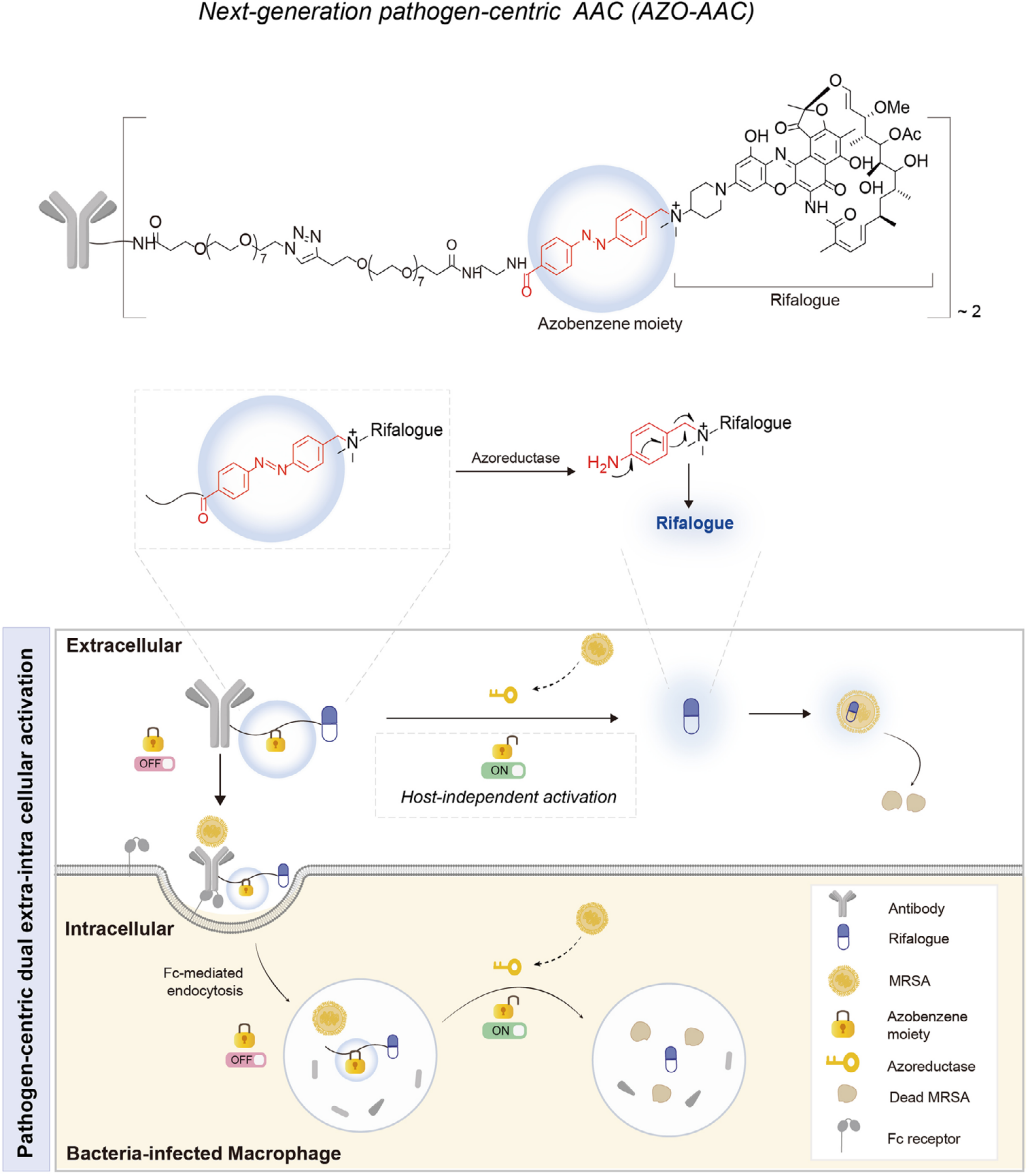
Schematic illustration of the azoreductase‐responsive AAC. The azobenzene linker is cleaved by azoreductase secreted by MRSA, leading to the release of the Rifalogue payload. This bacteria‐enzyme‐triggered activation enables site‐specific drug delivery to the infection site and mediates the eradication of both intracellular and extracellular bacteria, independent of host‐cell lysosomal integrity. The chemical structure includes a chloride ion associated with the quaternary ammonium salt.

In this study, we designed a pathogen‐responsive AAC strategy that shifts the activation trigger from the host‐cell machinery to the pathogen itself. Although azoreductases are highly expressed in tumor cells and widely utilized as a trigger in many key areas, such as macromolecular drug carriers [26], metal–organic frameworks [27], theranostic agents [28], and ADCs for tumor therapy [29], to the best of our knowledge, how to incorporate bacterial azoreductase into AAC design remains unreported. By incorporating an azobenzene moiety responsive to bacterium‐specific azoreductase into the linker, we have engineered an AAC (AZO‐AAC) whose activation is independent of host lysosomes. This pathogen‐centric design enables antibiotic release within the infected niche and provides activity against both intracellular reservoirs and planktonic MRSA. In addition, the azoreductase‐responsive linker of AZO‐AAC enabled efficient clearance of MRSA across intracellular and extracellular niches (Figure 1). Moreover, in murine septicemia and peritonitis models, antibody‐guided targeting with the pathogen‐driven activation efficiently eliminated both acute and persistent MRSA infections, providing a robust blueprint for the future development of novel anti‐infectives.

## 2 Results

### 2.1 Rational Design of a Bacterial Enzyme Responsive AAC

**FIGURE 2:**
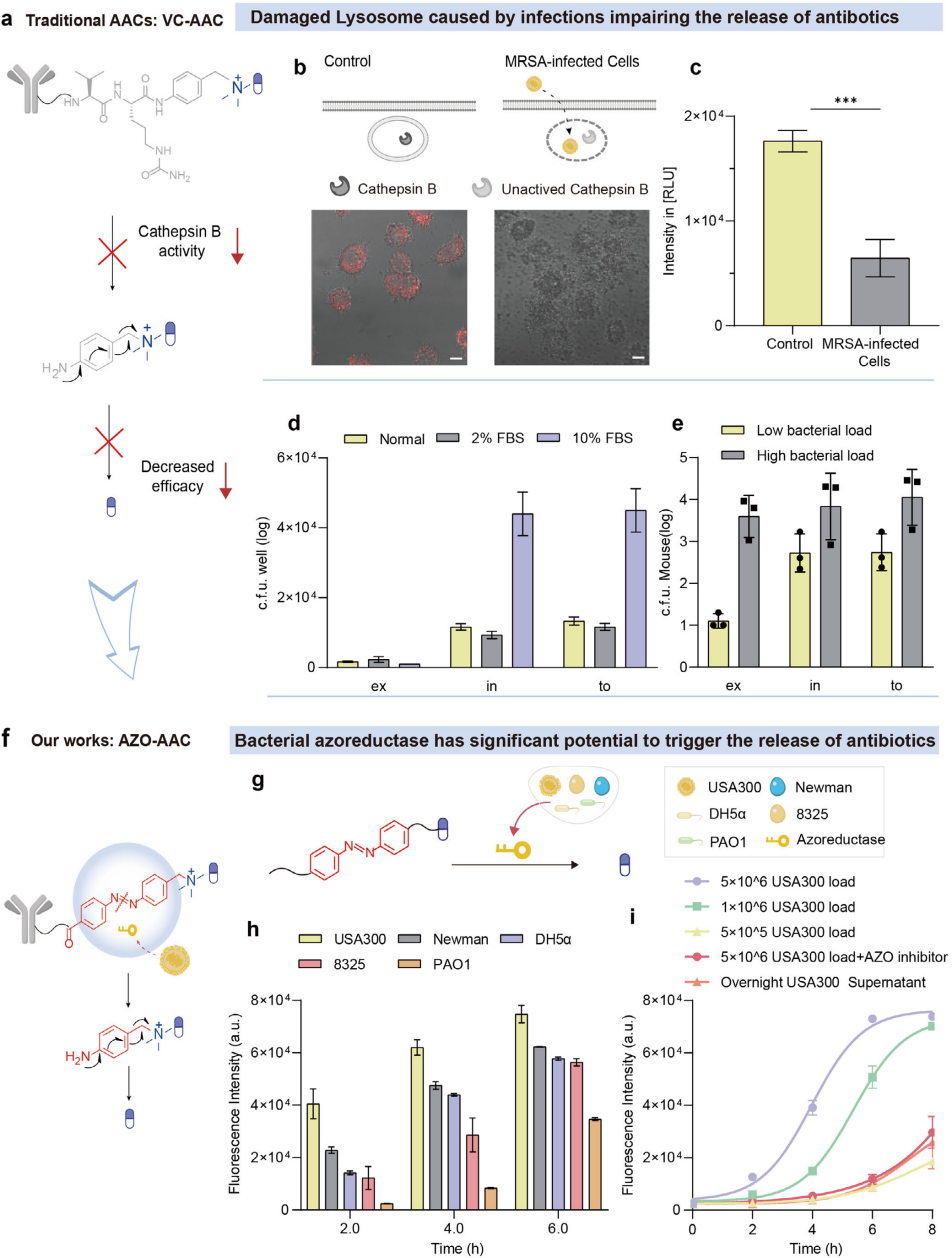
Design of the AACs based on enzyme‐triggered antibiotic release. (a) Traditional AACs rely on the release of CTSB from host cells lysosomes to trigger antibiotic release. (b) Confocal microscopy analysis of the lysosomal environment in an intracellular bacterial model; lysosomal CTSB activity was measured using Magic Red. Scale bar = 10 µm. (c) Cathepsin fluorescent substrate assay for detection of cathepsin cleavage. (d) Different concentrations of FBS were employed to simulate varying bacterial proliferation rates. Antimicrobial efficacy of AACs in Raw 264.7 cells. (e) Antibacterial efficacy of AACs in murine models with varying bacterial burdens. (f) AZO‐AAC releases antibiotics in response to bacterial‐specific azoreductases. (g) Schematic illustration of multiple azoreductase‐secreting bacterial strains capable of triggering antibiotic release. Fluorescence intensity was measured with a high‐throughput microplate reader to continuously monitor the enzymatic hydrolysis of the substrate (excitation wavelength 370 nm; emission wavelength 460 nm). Bacterial cultures or culture supernatants containing 5 × 10^5^ to 5 × 10^6^ CFU per reaction were co‐incubated with BioTracker 520 azoreductase sensor (Bz‐FVR‐AMC) at 37°C for different times to a final concentration of 1 µm. (h) Azoreductase expression in different bacterial strains. (i) Mimicking antibiotic release using an azobenzene moiety‐containing probe under different MRSA bacterial loads over time. For (d,e,h,i) are presented as mean ± SD from three independent experiments (*n* = 3).

Traditional antibody‐antibiotic conjugates (AACs) employ a dipeptide‐based linker (e.g., Val‐Cit/Ala) design borrowed from antibody‐drug conjugates (ADCs) [10, 30, 31, 32], which depends on host‐lysosomal proteases for antibiotic release (Figure 2a). However, during bacterial infection, host‐lysosomal function is frequently disrupted [14, 15], thereby limiting antibiotic release efficiency (Figure 2a). Confocal imaging corroborated that bacterial infection induces LMP (Figure S1b) [14, 15], disrupts intralysosomal pH homeostasis (Figure S1a) [33, 34], and markedly suppresses protease activity (Figure 2b,c) [18]. To establish a direct correlation between bacterial growth kinetics and host‐lysosomal dysfunction, varying concentrations of fetal bovine serum (FBS) were employed to simulate different bacterial proliferation rates. We observed that accelerated bacterial proliferation inversely correlated with the antimicrobial efficacy of traditional AACs (Figure 2d). This observation was further validated in vivo, as the initial bacterial burden in mice increased, AAC efficacy was significantly attenuated (Figure 2e). These findings indicate that the host‐centric design of current AACs is fundamentally constrained in the context of acute infections, underscoring the urgent need for pathogen‐responsive alternatives.

Notably, bacteria secrete a variety of enzymes, such as *β*‐lactamases, nitroreductases, azoreductases, and quinone reductases, that have been exploited as activation triggers for intelligent antibacterial delivery platforms, including pathogen‐centric AACs [25, 35]. Thus, previous works suggested to us that these bacterial‐specific enzymes could be harnessed to trigger targeted antibiotic release [36]. Specifically, bacterial azoreductase is known for its ability to efficiently and selectively cleave azobenzene moieties [26, 37]. Inspired by this, we developed a novel AAC platform (AZO‐AAC) by incorporating an azoreductase‐responsive azobenzene linker (Figure 2f). This pathogen‐centric strategy decouples drug activation from host cellular machinery, potentially enabling dual eradication of intracellular and extracellular infections. To validate this concept, we confirmed the constitutive expression of azoreductase across multiple strains, including MRSA strain USA300, *S. aureus* strain Newman and 8325, *Escherichia coli* (*E.coli*) strain DH5α, and *Pseudomonas aeruginosa* (*P.aeruginosa)* strain PAO1, using a fluorogenic probe Bz‐FVR‐AMC (Figure 2g,h). Furthermore, antibiotic release was found to be bacterial load–dependent and could be abolished by the azoreductase inhibitor diphenyliodonium (DPI), confirming that linker cleavage is specifically mediated by the pathogen (Figure 2i). Collectively, these results establish the azobenzene‐based AAC as a robust and promising platform for dual‐targeted antimicrobial therapy.

### 2.2 Synthesized AZO‐AAC Exhibits Excellent Stability and Efficient Drug Release

**FIGURE 3:**
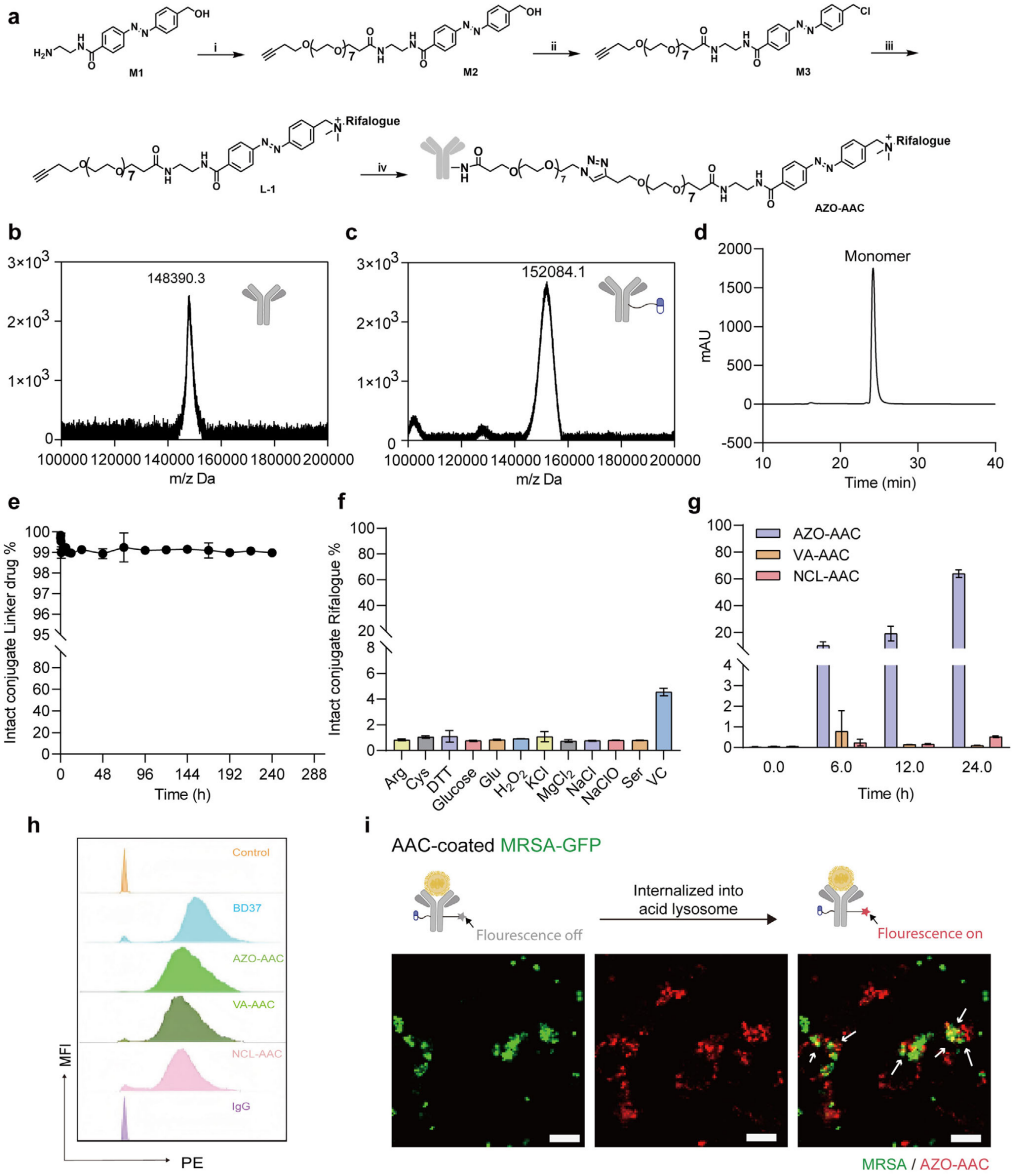
AZO‐AAC meets the quality control standards for AACs. (a) Synthesis route of AZO‐AAC. Experimental conditions: (i) DMF, DIPEA, rt, overnight; (ii) Cyanuric chloride, DIPEA, DMF, rt, 5 h; (iii) Rifalogue, DIPEA, DMF, rt, 6d; (iv) CuSO4, THPTA, Sodium ascorbate, DMA, HEPES, rt, 12–24 h. (b,c) MALDI‐TOF analysis of BD37 and AZO‐AAC. (d) SEC analysis of AZO‐AAC. (e) The stability of AZO‐AAC in human sera. (f) In vitro Rifalogue release in the presence of different physiological ions (Arg: *L*‐Arginine, Cys: *L*‐Cysteine, DTT: Dithiothreitol, Glucose: *D*‐Glucose, Glu: *L*‐Glutamic acid, H_2_O_2_: Hydrogen peroxide, KCl: Potassium chloride, MgCl_2_: Magnesium chloride, NaCl: Sodium chloride, NaClO: Sodium hypochlorite, Ser: *L*‐Serine, VC: Ascorbic acid). (g) In vitro Rifalogue release in the presence of azoreductase under MRSA conditions. (h) Flow cytometry analysis of BD37 and AZO‐AAC binding to *β*‐WTA antigens expressed by MRSA. (i) Confocal microscopy analysis of internalization of USA300‐GFP and AZO‐AAC complex. Scale bar = 5 µm. For (e–h) data are presented as mean ± SD from three independent experiments (*n* = 3).

The synthetic route for AZO‐AAC is illustrated in Figure 3a. Compound M1 was synthesized according to reported procedures [29]. To mitigate the inherent hydrophobicity of the azobenzene moiety, poly(ethylene glycol) (PEG) spacers were incorporated into the linker to improve aqueous solubility, yielding M2. Subsequent chlorination of M2 followed by nucleophilic substitution with Rifalogue afforded the alkyne‐functionalized linker L‐1. The final AZO‐AAC was obtained via copper‐catalyzed azide‐alkyne cycloaddition (CuAAC) between L‐1 and an azide‐functionalized neutralizing anti‐*S. aureus* antibody (BD37), this antibody only targets *S. aureus*. For comparative studies, we also synthesized a traditional protease‐cleavable conjugate (VA‐AAC) and a non‐cleavable conjugate (NCL‐AAC) as a negative control (Figure S2). The identity of L‐1 was confirmed by high‐resolution mass spectrometry (^1^H‐NMR), and the DAR values for all conjugates were determined by MALDI‐TOF MS (see Supporting Information).

We next characterized the physicochemical properties of AZO‐AAC to ensure it met the stringent requirements for therapeutic conjugates, including optimized DAR, high monodispersity, and serum stability. MALDI‐TOF analysis confirmed successful bio‐conjugation, showing a mass shift from 148 390.3 to 152 084.1 Da, consistent with an average DAR of approximately 2 (Figure 3b,c). Size‐exclusion chromatography (SEC) verified that AZO‐AAC maintained a high monomeric fraction with minimal aggregation (<5%) (Figure 3d). Crucially, AZO‐AAC exhibited exceptional stability in circulation. Incubation in human serum for 10 days resulted in negligible premature release (<5%) (Figure 3e), and common physiological ions did not trigger antibiotic release. Although VC (sodium ascorbate) as a reducing agent may have contributed to a slight increase, the premature release of Rifalogue remained minimal (not exceeding 6%) (Figure 3f). In contrast, incubation with MRSA triggered the time‐dependent release of Rifalogue (∼60% at 24 h), which was mediated by bacterial azoreductase (Figure 3g). The fact that VA‐AAC and NCL‐AAC remained stable under the same conditions confirms that azoreductase is indispensable for the activation of our pathogen‐responsive linker.

To confirm that conjugation did not compromise target engagement, flow cytometry was employed to evaluate the binding affinity of AZO‐AAC toward MRSA. The conjugation retained binding efficacy comparable to the parental BD37 antibody, ensuring that the modification of the Fc region (via azide functionalization) did not alter antigen specificity (Figure 3h). Finally, we visualized the intracellular trafficking of AZO‐AAC using a dual‐fluorescence imaging assay. GFP‐expressing MRSA (USA300‐GFP) opsonized with pH rodo^TM^‐labeled AZO‐AAC were efficiently internalized by macrophages. The intense fluorescence observed within acidic lysosomes confirmed successful co‐localization and internalization of the conjugate with its bacterial target (Figure 3i). Collectively, these results demonstrate the successful synthesis of a stable, well‐defined AZO‐AAC that undergoes specific, pathogen‐dependent activation while retaining full targeting capacity.

### 2.3 AZO‐AAC Demonstrates Superior Efficacy Against Planktonic and Intracellular MRSA In Vitro

**FIGURE 4:**
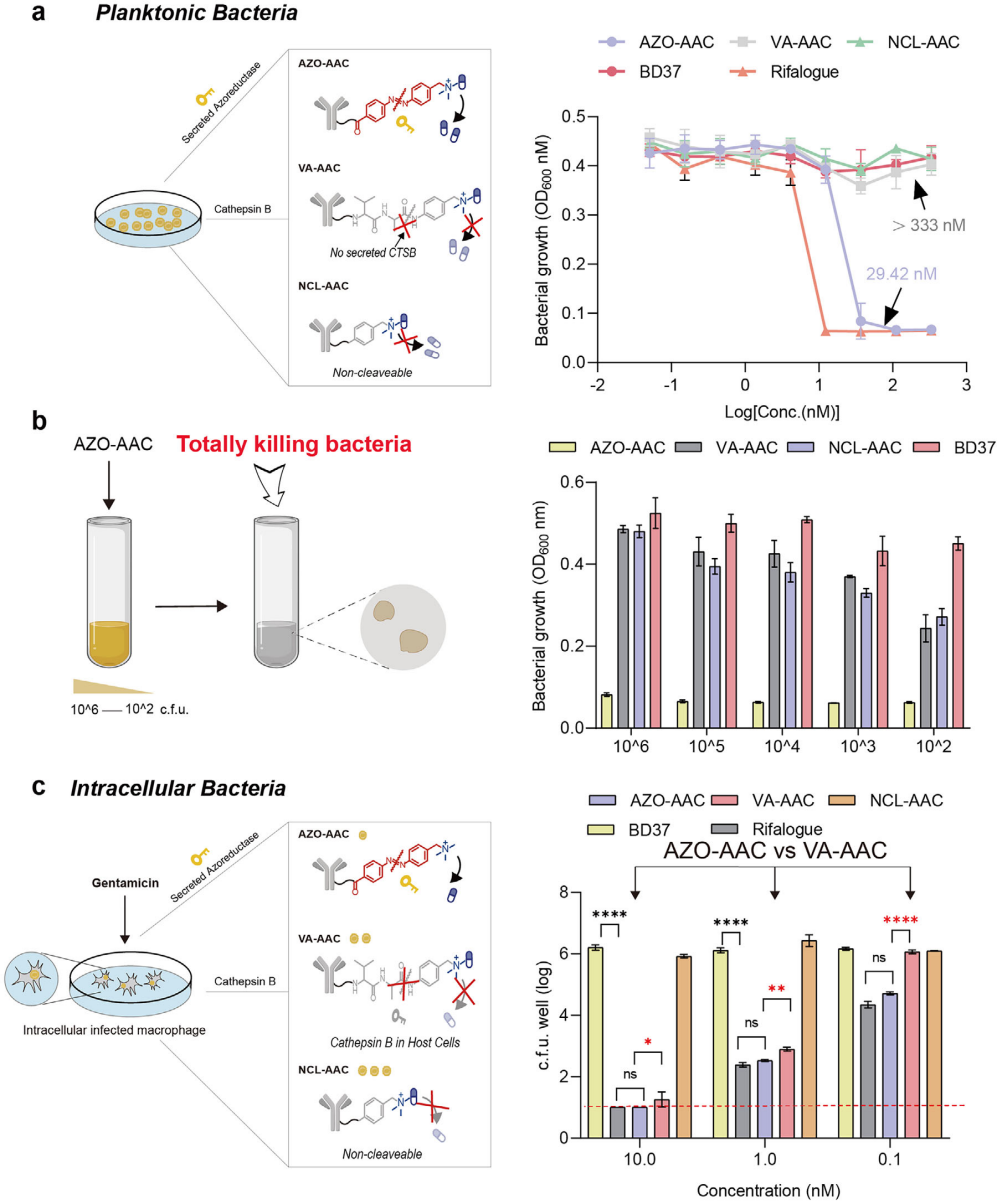
Antibacterial activity of AZO‐AAC for planktonic and intracellular bacteria. (a) MIC determination for Rifalogue, BD37, and AACs against MRSA. (b) Survival of stationary‐phase MRSA incubated with 50 µg·mL^−^^1^ AACs. (c) MRSA opsonized with BD37, Rifalogue, or AACs was enumerated 24 h after incubation with the indicated cell lines. The horizontal dashed line indicates the limit of detection. Data are presented as mean ± SD from three independent experiments (*n* = 3). Statistical analyses were performed using ordinary one‐way ANOVA with multiple comparisons. Significance levels are indicated as follows: ^*^ *p* < 0.05, ^**^ *p* < 0.005, ^***^ *p* < 0.0002, ^****^ *p* < 0.0001, ns, not significant. The figure was created with BioRender.com, with permission.

We next evaluated the antimicrobial efficacy of the AZO‐AAC compared with relevant controls, including VA‐AAC, NCL‐AAC, free Rifalogue, and BD37 antibody. Notably, in the planktonic growth inhibition assay, AZO‐AAC retained the antibiotic activity of Rifalogue, exhibiting potent activity (MIC = 29.4 nm). Its activity was more than tenfold greater than that of VA‐AAC, NCL‐AAC, and BD37 antibody, all of which showed MIC values >333 nm (Figure 4a). This result directly validates our design principle: the bacterial enzyme‐triggered mechanism enables efficient antibiotic release in the extracellular environment where azoreductase is present, thereby allowing AZO‐AAC to effectively target and eliminate free‐living bacteria. AZO‐AAC achieved complete sterilization within 16–18 h, demonstrating potent bactericidal activity across different initial bacterial loads (Figure 4b).

Subsequently, we evaluated the ability of this conjugation to eliminate MRSA within macrophages. Consistent with our design expectations, AZO‐AAC exhibited dose‐dependent killing of intracellular bacteria, with efficacy matching that of free Rifalogue at equivalent concentrations (Figure 4c). Notably, VA‐AAC exhibited significantly reduced activity at various drug concentrations in this model, potentially due to impaired CTSB activity caused by MRSA infection (as shown in Figure 2). As expected, the NCL‐AAC and BD37 antibody alone showed no appreciable antibacterial effect, confirming that targeted antibiotic release is essential for activity (Figure 4c).

### 2.4 AZO‐AAC Achieves Unparalleled Efficacy in a Severe Both Intra‐ and Extracellular Infection In Vitro

**FIGURE 5:**
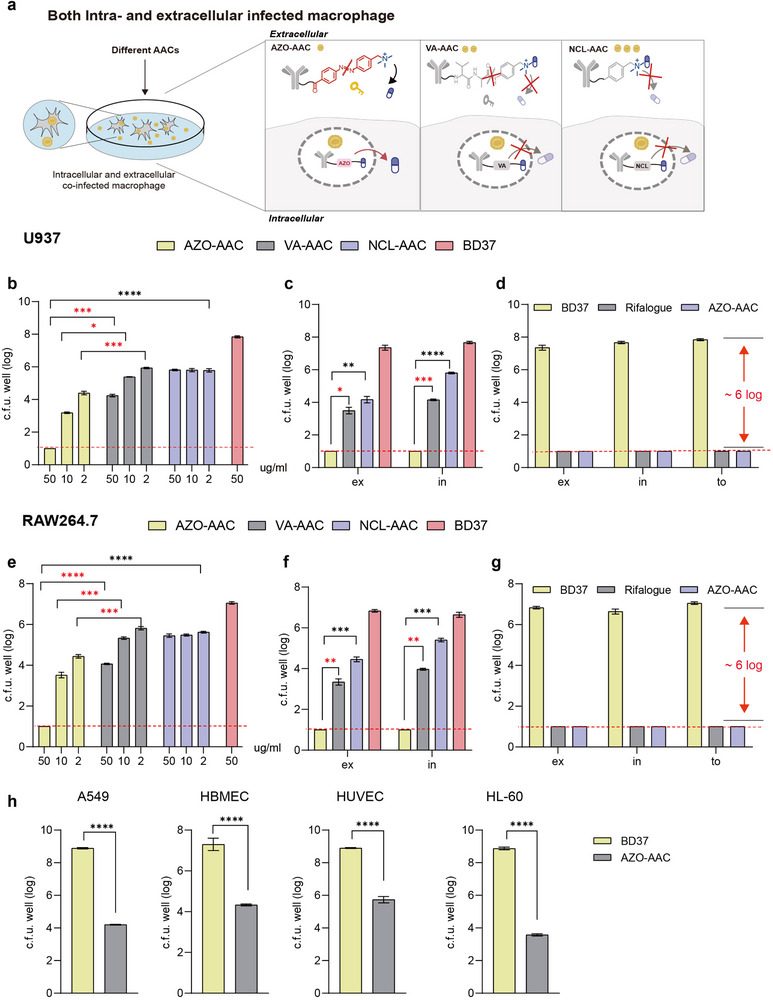
Antimicrobial activity of AACs in a combined intracellular and extracellular bacterial infection model. (a) Schematic illustration of the combined intracellular and extracellular infection model and the proposed mechanisms of action of AACs under these conditions. (b) Antimicrobial efficacy of AACs in U937 cells. (c) Separate antibacterial activities of AACs against intracellular and extracellular bacteria in U937 cells. (d) Comparison of the antibacterial activity of AZO‐AAC and Rifalogue in U937 cells. (e) Antimicrobial efficacy of AACs in Raw 264.7 cells. (f) Separate antibacterial activities of AACs against intracellular and extracellular bacteria in Raw 264.7 cells. (g) Comparison of the antibacterial activity of AZO‐AAC and Rifalogue in Raw 264.7 cells. (h) Antimicrobial effect of AZO‐AAC across different cell lines. The horizontal dashed line indicates the limit of detection. The data in b and e were further analyzed based on (c,f), respectively. Data are presented as mean ± SD from three independent experiments (*n* = 3). Statistical analyses were performed using ordinary one‐way ANOVA with multiple comparisons. Significance levels are indicated as follows: ^*^ *p* < 0.05, ^**^ *p* < 0.005, ^***^ *p* < 0.0002, ^****^ *p* < 0.0001, ns, not significant.

The antibacterial activities of AZO‐AAC were evaluated in the aforementioned planktonic and intracellular infection models. However, the actual state of infection involves bacteria coexisting both intra‐ and extracellularly. To determine the activity of AZO‐AAC in the actual state of infection, we established a more severe both intracellular and extracellular infection model (Figure 5a). This model enables simultaneous quantification of antibacterial efficacy against intracellular and extracellular bacteria within the same system. In this stringent model, AZO‐AAC demonstrated exceptionally potent bactericidal activity in a dose‐dependent manner, regardless in human macrophages U937 cells or mouse macrophages Raw 264.7 cells (Figure 5b,e; Figure S3a). Especially at the dose of 50 µg mL^−1^, AZO‐AAC achieved the complete elimination of MRSA, with a potency comparable to that of free Rifalogue (Figure 5d,g). In contrast, VA‐AAC exhibited minimal activity (∼3 log_10_ units), whereas NCL‐AAC showed ∼5 log_10_ units.

Subsequently, the antibacterial efficacy of AZO‐AAC was analyzed separately against intracellular and extracellular bacteria. At a concentration of 50 µg mL^−^^1^, AZO‐AAC reduced the MRSA burden below the limit of detection in both compartments (Figure 5c,f; Figure S3b). This dual efficacy is a direct consequence of its bacterial enzyme‐triggered mechanism, which operates independently of host‐cell status. In striking contrast, VA‐AAC showed markedly reduced efficacy, particularly against intracellular bacteria, further indicating that AACs relying on lysosomal protease are suboptimal in this infection context. As expected, NCL‐AAC and BD37 antibodies were virtually inactive. These results validate the rationale for employing a bacterial enzyme‐responsive linker, which is uniquely suited to combat infections in real‐world pathological settings. In addition, AZO‐AAC could also kill MRSA in every cell type tested, including macrophages (Raw 264.7, U937), endothelial cells (HBMEC, HUVEC), epithelial cells (A549), and neutrophils (HL‐60) (Figure 5h), highlighting its broad therapeutic potential for treating infections in diverse tissues.

### 2.5 AZO‐AAC Exhibited Excellent In Vivo Targeting of Infection Sites

**FIGURE 6:**
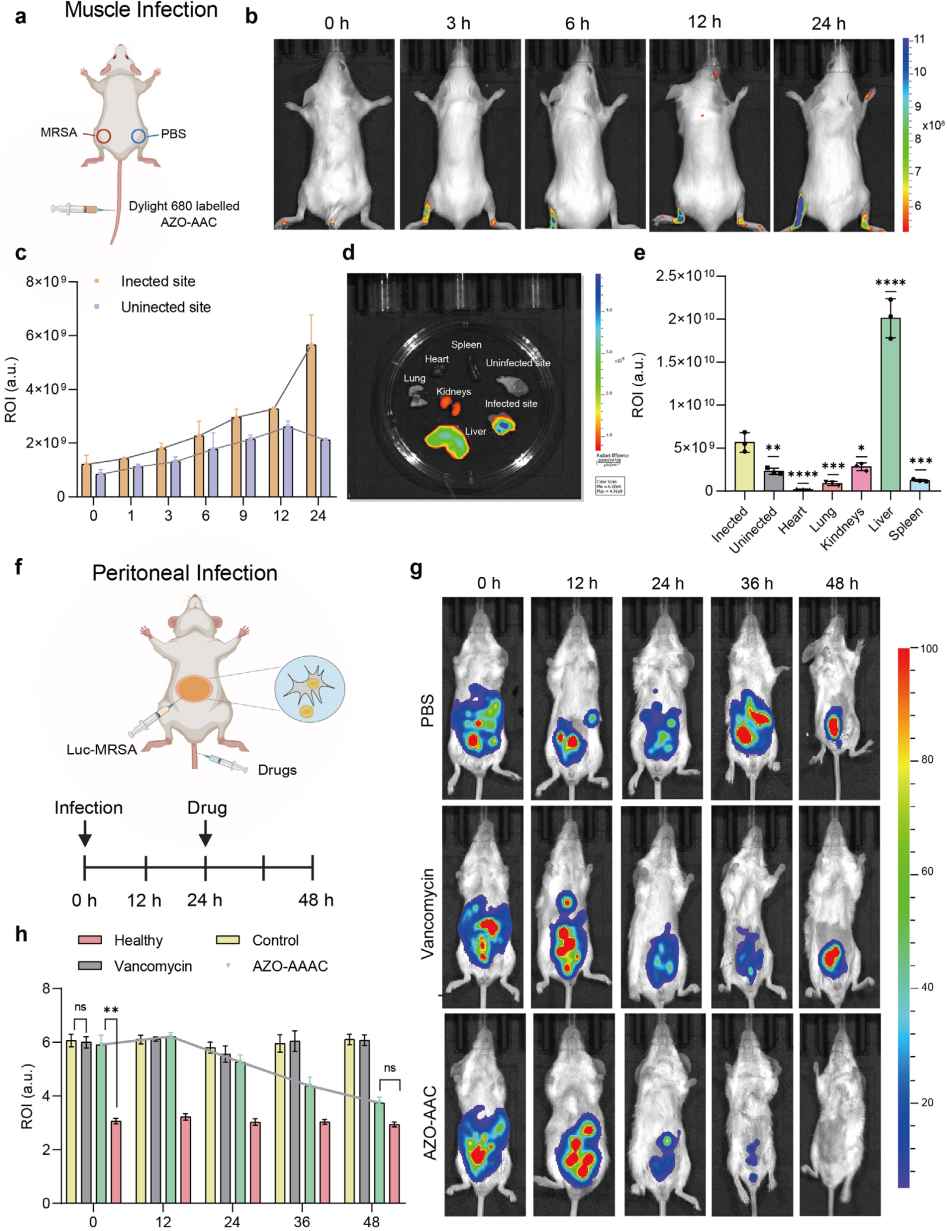
In vivo targeting and preliminary efficacy of AZO‐AAC. (a) Schematic illustration of the muscle infection model. (b) The fluorescence images of the infected mice at the indicated time points. (c) Quantitative analysis of the ROI analysis of the infected and the uninfected sites, *n* = 3. (d) Ex vivo fluorescence images of the major organs and tissues collected from the MRSA‐infected mice after 24 h i.v. injection. (e) Quantitative analysis of the ROI analysis of the organs or tissues in the ex vivo images, *n* = 3. (f) Schematic illustration of the peritonitis infection model. (g) Quantitative analysis of the ROIs in the peritoneal cavity of mice in different treatment groups, *n* = 5. (h) Fluorescence images of infected mice at different time points under various drug treatments. Data are presented as mean ± SD from three independent experiments (*n* = 3/5). Statistical analyses were performed using ordinary one‐way ANOVA with multiple comparisons. Significance levels are indicated as follows: ^*^ *p* < 0.05, ^**^ *p* < 0.005, ^***^ *p* < 0.0002, ^****^ *p* < 0.0001, ns, not significant. The figure was created with BioRender.com, with permission.

Optical in vivo imaging is a commonly used method for verifying drug targeting. Therefore, we employed this approach in a murine model of localized MRSA infection. DyLight 680‐labeled AZO‐AAC was administered intravenously to murine subjects 24 h after intramuscular injection of MRSA into the right thigh muscle (infected site) and PBS into the left thigh muscle (uninfected site) (Figure 6a). In vivo imaging revealed robust, time‐dependent accumulation of the AZO‐AAC specifically at the infected site, with a strong fluorescence signal still observed at 24 h post‐injection, the final imaging time point (Figure 6b,c). Ex vivo imaging of harvested organs and tissues at 24 h further confirmed this targeted distribution, showing significant conjugate accumulation in the infected muscle, alongside expected clearance organs (liver and kidneys) (Figure 6d). Critically, quantification of the region of interest (ROI) demonstrated a greater than two‐fold higher accumulation at the infected site compared to the uninfected control site (^***^ *p* = 0.0048) (Figure 6e). This selective enrichment provides direct visual evidence that the AZO‐AAC effectively homes to the infectious focus.

To further validate the targeting efficiency of AZO‐AAC, we employed bioluminescent MRSA (USA300‐*luc*) to dynamically monitor bacterial clearance following AZO‐AAC accumulation at the infection site (Figure 6f). At 12 h post‐infection, robust luminescence confirmed successful establishment of the model. After a single intravenous dose administered at the 24 h time point, the AZO‐AAC treatment group exhibited a rapid and sustained reduction in bacterial luminescence. By the 48 h endpoint, the AZO‐AAC group achieved a state of bacterial clearance that was statistically indistinguishable from that in healthy, uninfected controls (Figure 6g,h). This near‐complete eradication underscores the profound efficacy of the azoreductase‐triggered antibiotic release of AZO‐AAC.

Collectively, these in vivo results demonstrate that the AZO‐AAC platform not only enables targeted drug delivery but also mediates in vivo inhibition of bacteria, translating into superior therapeutic efficacy in murine models of both intracellular and extracellular MRSA infection.

### 2.6 AZO‐AAC Demonstrated Significant Antibacterial Efficacy in an In Vivo Septicemia Model

**FIGURE 7:**
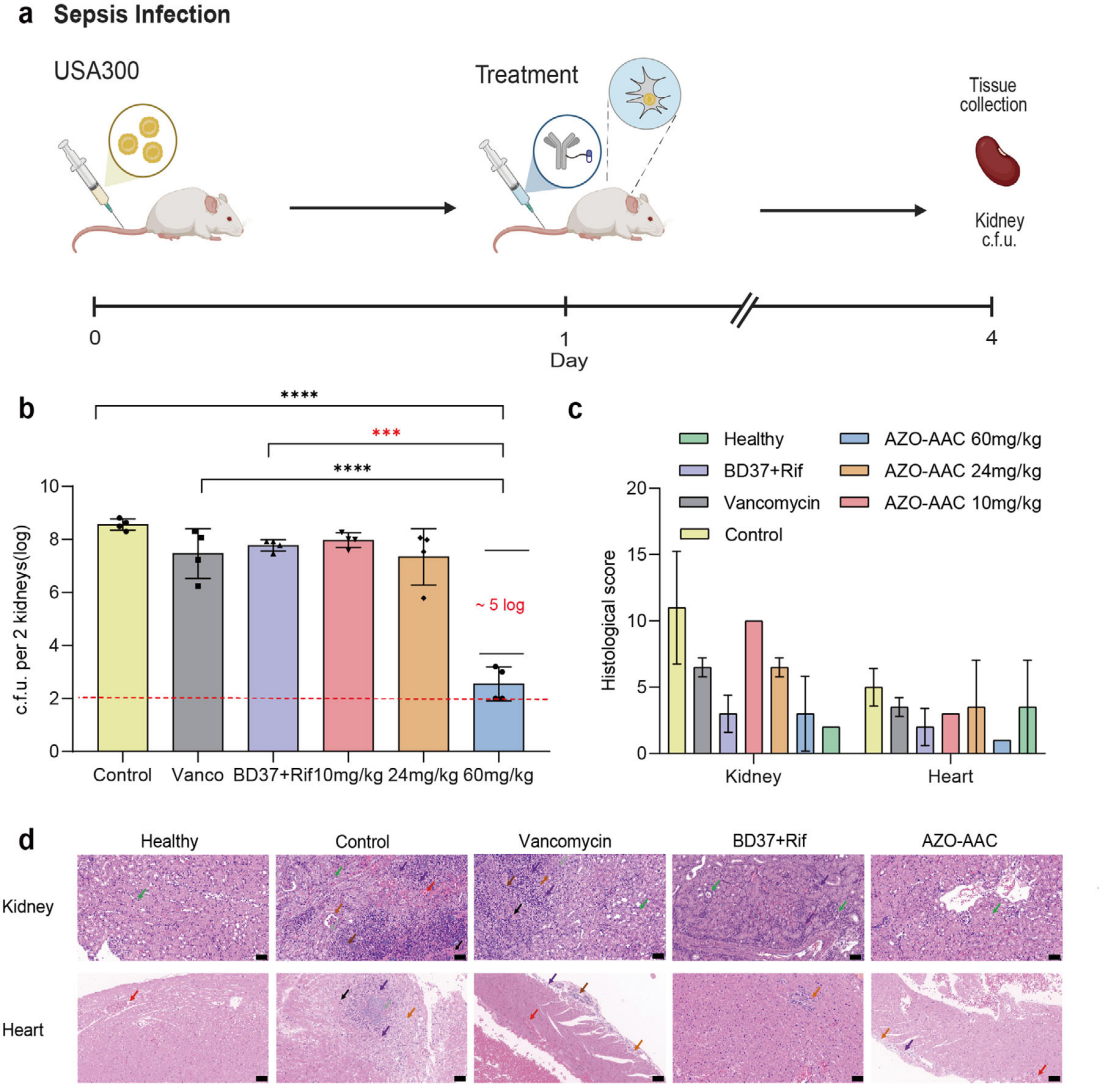
Antibacterial activity of AZO‐AAC in a murine model of septicemia. (a) Schematic illustration of the septicemia model. (b) Kidneys were homogenized, and bacterial burden was determined via smear analysis followed by statistical evaluation, *n* = 4. (c) H&E scoring was assigned to various mouse tissues. (d) Representative H&E‐stained sections of the kidney and heart from different treatment groups. Black arrow: Extensive foci of necrosis; Purple arrow: Nucleolar fragmentation with homogeneous eosinophilic staining, accompanied by abundant lymphocytic and granulocytic infiltration; Brown arrow: Visible basophilic masses, suspected fungal clusters. Scale bar = 50 µm. The horizontal dashed line indicates the limit of detection. Data are presented as mean ± SD from three independent experiments (*n* = 4). Statistical analyses were performed using ordinary one‐way ANOVA with multiple comparisons. Significance levels are indicated as follows: ^*^ *p* < 0.05, ^**^ *p* < 0.005, ^***^ *p* < 0.0002, ^****^ *p* < 0.0001, ns, not significant. The figure was created with BioRender.com, with permission.

Next, we conducted in vivo efficacy assessments using the original MRSA strain. We first established a septicemia model in which bacteria coexist both intracellular and extracellularly. At this stage, bacteria often colonize the kidneys, making the kidneys bacterial burden the gold standard for evaluating drug efficacy in this model [38]. Vancomycin, serving as the last line of defense against clinical infections, proved ineffective at eradicating intracellular bacteria in this model [39] (Figure 7a). Administration of AZO‐AAC (60 mg kg^−^^1^) led to a profound reduction in the kidneys' bacterial burden, with more than a ~5 log_10_ decrease compared with the untreated group (Figure 7b). This antibacterial efficacy significantly surpassed that of the clinical standard‐of‐care vancomycin, as well as BD37 and free Rifalogue. The superior performance relative to the antibody‐antibiotic combination highlights the critical role of the bacterial enzyme‐responsive activation mechanism in delivering high local concentrations of antibiotic directly at the infection site.

Most importantly, the potent bactericidal activity of AZO‐AAC translated into remarkable histopathological outcomes. Histological analysis by hematoxylin and eosin (H&E) staining of kidneys and hearts from infected murine in the drug‐treated and control groups, as well as from healthy murine demonstrated severe infection‐associated damage, including extensive necrosis, dense inflammatory infiltration, interstitial fibrosis, tubular atrophy, and suspected bacterial clusters (Figure 7d). In stark contrast, tissues from murine treated with the high‐dose AZO‐AAC were histologically indistinguishable from those of healthy, uninfected controls. This profound tissue‐protective effect was further corroborated by semi‐quantitative H&E scoring, which revealed in the AZO‐AAC‐treated group, histopathology of the kidney and heart histopathology in the AZO‐AAC‐treated group was comparable to that of normal murine (Figure 7c).

Collectively, these results demonstrate that AZO‐AAC not only substantially reduces bacterial burden but also preserves organ integrity and function following lethal systemic infection, thereby providing potent protection against systemic MRSA infection and its pathological consequences.

### 2.7 AZO‐AAC Eradicated Bacteria Both Intracellular and Extracellular Bacteria in an *I* *n Vivo* Peritonitis Model

To rigorously evaluate the therapeutic potential of AZO‐AAC, we employed a murine model of peritonitis by MRSA infection featuring the dual challenge of intra‐ and extracellular infection (Figure S4a). AZO‐AAC exhibited a potent, dose‐dependent reduction in bacterial load across all compartments (Figure S4b,c). The efficacy plateau at 60 mg kg^−^^1^ and above established this as the dose sufficient to achieve maximal target engagement and bacterial clearance, defining a clear therapeutic index for subsequent studies.

The true power of our bacterial enzyme‐activated design became particularly evident when we challenged a key limitation of its host‐activated conjugation (VA‐AAC). We observed that the efficacy of VA‐AAC became markedly attenuated under conditions of high bacterial burden, whereas AZO‐AAC was entirely unaffected (Figure S5b). This phenomenon may be attributed to the MRSA‐induced impairment of lysosomal function, which compromises the CTSB‐dependent activation mechanism of VA‐AAC. In contrast, AZO‐AAC operates independently of the host‐cell machinery. This resilience was further supported by the persistence of intracellular bacteria at lower infection doses, where antibiotic release from AACs proved insufficient to eradicate intracellular reservoirs (Figure S5a).

**FIGURE 8:**
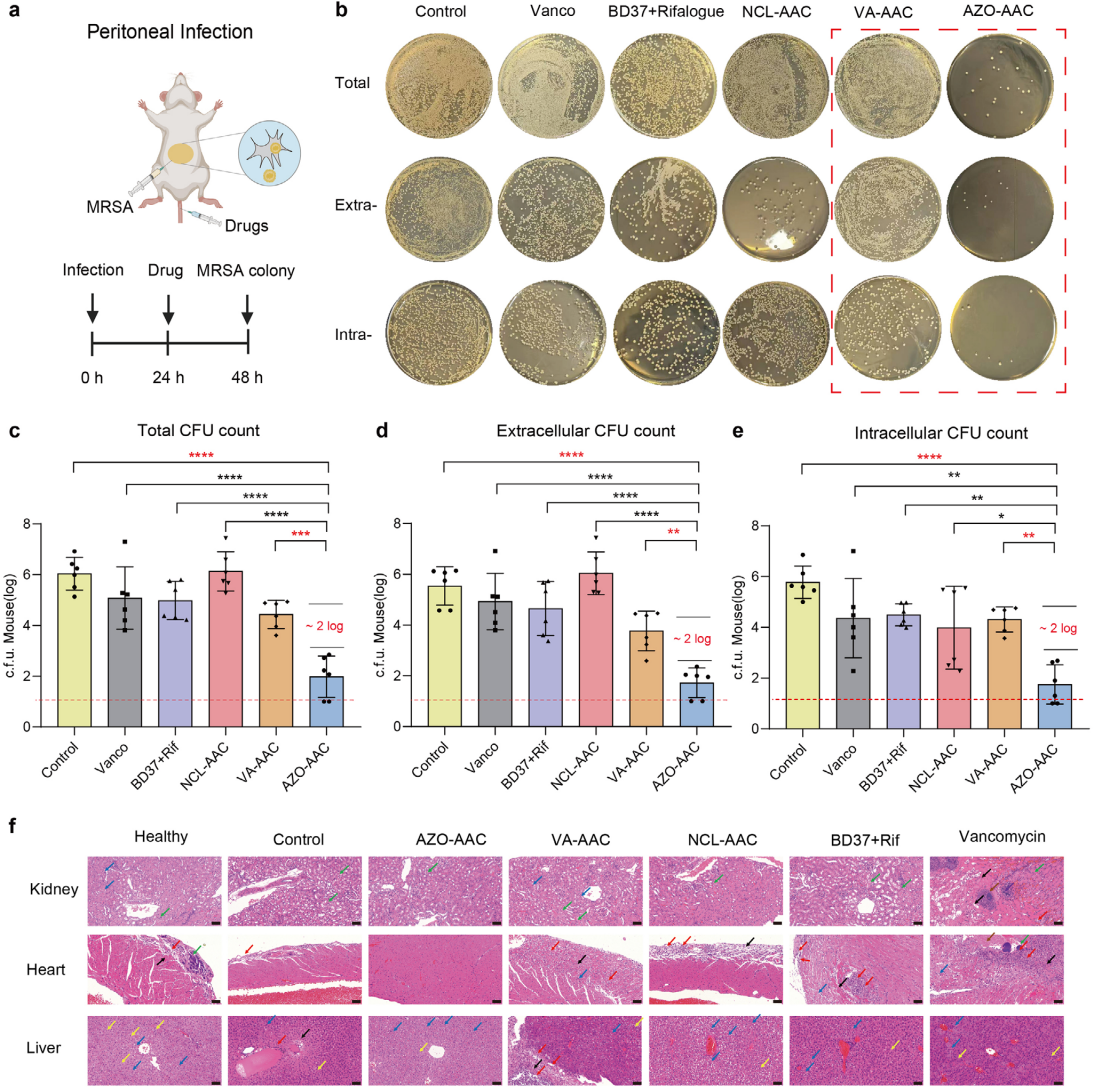
Antibacterial activity of AZO‐AAC in a peritoneal model. (a) Schematic illustration of the peritonitis model. (b) Representative CFUs images from infected mice under different treatments. (c) Total CFUs were determined 24 h after the different treatments (*n* = 6). (d) Extracellular CFUs were determined 24 h after the different treatments (*n* = 6). (e) Intracellular CFUs were determined 24 h after the different treatments (*n* = 6). (f) Representative sections of heart, kidney, and liver from different treatments stained with H&E. Black arrow: Extensive focal necrosis; Red arrow: Accompanied by nuclear fragmentation and eosinophilic staining, with focal lymphocytic and granulocytic infiltration. Scale bar = 50 µm. The horizontal dashed line indicates the limit of detection. Data are presented as mean ± SD from three independent experiments (*n* = 6). Statistical analyses were performed using ordinary one‐way ANOVA with multiple comparisons. Significance levels are indicated as follows: ^*^ *p* < 0.05, ^**^ *p* < 0.005, ^***^ *p* < 0.0002, ^****^ *p* < 0.0001, ns, not significant. The figure was created with BioRender.com, with permission.

Encouraged by these findings, we next evaluated the in vivo antimicrobial efficacy of the AZO‐AAC relative to relevant controls, including the VA‐AAC, NCL‐AAC, BD37, and free Rifalogue and the clinical standard vancomycin in a high‐burden infection model. The results were unequivocal: AZO‐AAC achieved near‐complete eradication of the total bacterial burden, outperforming all other therapeutic strategies (Figure 8b). Dissection of the infection into its intracellular and extracellular components revealed the basis for this superiority (Figure 8c). While extracellular clearance was effectively achieved by all antibody‐based regimens, AZO‐AAC was the only construct capable of producing potent, statistically significant depletion of the resilient intracellular MRSA population (> 2 log_10_ units vs. VA‐AAC; Figure 8d). This finding provides definitive in vivo proof that the bacterial enzyme‐responsive cleavage mechanism remains fully functional within the hostile environment of an infected cell, a capability that is not shared by host‐dependent AACs whose activation is compromised (>2 log_10_ units vs. VA‐AAC; Figure 8e).

Unsurprisingly, histopathology analysis showed that kidney and heart tissues from AZO‐AAC‐treated murine were comparable to those of healthy controls. Upon blinded pathological evaluation confirmed that tissues from AZO‐AAC‐treated murine were indistinguishable from those of healthy controls. For comparison, the control group exhibited severe multi‐organ injury consistent with progressive of septicemia (Figure 8f; Figure S6).

In summary, this study provides compelling evidence that our bacterial enzyme‐responsive AAC platform represents a transformative therapeutic strategy. It uniquely resolves compartmentalized infections, confers absolute organ protection, and sets a new benchmark for efficacy, surpassing both current standards and prior‐generation conjugate technologies.

## 3 Discussion

In this study, we established a pathogen‐responsive AAC platform predicated on bacterial enzyme‐mediated drug activation. The central innovation of this strategy lies in decoupling antimicrobial release from host cellular health by transitioning from host‐dependent activation to a pathogen‐centric paradigm. While traditional AACs—largely a paradigm inheritance from ADCs—have demonstrated value in targeted delivery [10, 40, 41, 42], they remain functionally tethered to the activity of host‐lysosomal proteases activity [10, 30, 31, 32]. Our findings reveal that during acute infection, MRSA‐induced LMP and the consequent disruption of pH homeostasis significantly suppress lysosomal protease activity, creating a therapeutic bottleneck for host‐dependent conjugates (as shown in Figure 2) [14, 15, 18, 33, 34].

Distinguishable from these traditional designs, AZO‐AAC shifts the activation trigger to bacterial‐secreted azoreductases. We demonstrated that AZO‐AAC achieves efficient payload release (60% over 24 h) in the presence of planktonic MRSA, whereas conventional conjugation remains largely dormant in the absence of host‐derived CTSB [10]. Sodium ascorbate (VC), as a reducing agent, induced only minimal release of the Rifalogue analogue (< 6%), suggesting that non‐enzymatic reduction can occur to a limited extent but is insufficient for efficient payload activation. This fundamental shift enables context‐independent drug release, maintaining therapeutic efficacy regardless of the pathogen localization or the metabolic status of host cells.

Consequently, AZO‐AAC overcomes the intrinsic limitations of current AACs in high‐burden infection settings and demonstrates superior bactericidal potency across multiple in vitro and in vivo infection models.

More broadly, leveraging the bacterial “enzymatic signature” represents a versatile strategy with broad applicability. Beyond azoreductases, various pathogen‐specific enzymes, such as *β*‐lactamases and nitroreductases [25, 40, 41, 42], could be harnessed to develop pathogen‐centric “intelligent” AACs tailored to specific pathogens. Nevertheless, the clinical translation of this platform will require addressing the spatiotemporal heterogeneity of bacterial enzyme expression, which may vary across different strains, growth phases (e.g., biofilm states), and infection micro‐environments. Furthermore, the pathogen‐dependent nature of this platform implies a potential threshold effect, whereby suboptimal antibiotic release may occur under conditions of extremely low bacterial burden. Future studies should focus on evaluating the synergistic potential of combining AZO‐AACs with first‐line clinical antibiotics to provide a more robust antimicrobial response.

In summary, by transferring the activation trigger to the pathogen itself, the AZO‐AAC platform provides a powerful solution for tackling recalcitrant intracellular infections. This work points toward a pivotal future direction in antimicrobial development: achieving precision therapy through intelligent, pathogen‐driven drug activation.

## 4 Materials and Methods

### 4.1 Ethics Statement

Female BALB/c Murine (6–8 weeks old) were obtained from Vital River Laboratories (Beijing, China) and maintained under specific pathogen‐free conditions. All animal procedures were conducted in accordance with protocols approved by the Animal Ethics Committee of the Beijing Institute of Pharmacology and Toxicology (Approval No. IACUC‐DWZX‐2020‐697) and were further reviewed and approved by the Health Research Ethics Committee of the Capital Region, China.

### 4.2 Bacterial Strains

Methicillin‐resistant *Staphylococcus aureus* USA300, USA300‐*luc*, and USA300‐GFP strains were obtained from Bio‐Sci Co., Ltd. (http://www.bio‐sci.com.cn) and used for all in vivo experiments unless otherwise specified. Additional strains, including *S. aureus* Newman, *S. aureus* 8325,* E. coli* DH5α, and *P. aeruginosa* PAO1(*WT*) were obtained from pre‐existing laboratory frozen stocks.

### 4.3 Verification of Azoreductase Expression

To quantify azoreductase activity, bacterial cultures at varying colony‐forming units (CFUs) (5 × 10^6^ CFU, 1 × 10^6^ CFU and 5 × 10^5^ CFU MRSA strain USA300, 5 × 10^6^ CFU *S. aureus* strain Newman and 8325, 5 × 10^6^ CFU *E.coli* strain DH5α and 5 × 10^6^ CFU *P.aeruginosa* strain PAO1 WT) or culture supernatants were incubated with BioTracker 520 Azoreductase Sensor (Bz‐FVR‐AMC) at a final concentration of 1 µm for 1 h at 37°C. Enzymatic cleavage of the substrate was continuously monitored using a high‐content microplate reader by measuring fluorescence intensity (excitation: 370 nm; emission: 460 nm). The release of fluorescent AMC was correlated with azoreductase activity levels.

### 4.4 Quality Control of AZO‐AAC—MALDI‐TOF Analysis

Molecular mass characterization was conducted using a Bruker Autoflex MALDI‐TOF mass spectrometer. Prior to analysis, samples were purified via SEC on a Shodex Protein KW‐802.5 column (8.0 mm × 300 mm, 5 µm) using PBS as the mobile phase at a flow rate of 0.5 mL/min. Acquired spectra were processed using FlexAnalysis 3.4 software.

### 4.5 Quality Control of AZO‐AAC—SEC Analysis

Aggregation state of AZO‐AAC was evaluated by SEC using an Agilent 1260 HPLC system equipped with a TSKgel G3000SWXL column (7.8 mm × 300 mm). The mobile phase consisted of 40 mm sodium phosphate and 150 mm sodium chloride (pH 7.0). Separation was performed isocratically at a flow rate of 1 mL/min, and detection was carried out by UV absorbance at 280 nm.

### 4.6 In Vitro Rifalogue Release Assay

To evaluate the stability and release profile of Rifalogue from the linker, compound L‐1 was prepared as a 5 mm stock solution in DMSO and diluted to 50 µm in PBS for release assays. The diluted conjugate was incubated under various conditions, including treatment with DTT, Arg, CaCl_2_, Cys, Glu, glucose, H_2_O_2_, KCl, MgCl_2_, NaCl, NaClO, Ser, or VC, with shaking at 37°C for 24 h. After incubation, all samples were quenched with cold methanol, centrifuged to remove precipitated proteins, and analyzed by LC‐MS/MS to quantify released Rifalogue.

### 4.7 Stability Testing of AZO‐AAC in Human Sera

The stability of AZO‐AAC was assessed in 50% human sera (Bioreclamation IVT). The conjugate was spiked into the sera to a final concentration of 200 nm and incubated at 37°C. Aliquots (100 µL) were collected at specified time intervals (0, 15, 30 min; 1, 3, 6, 9, 12 h; and 1–10 days). Each aliquot was immediately quenched with methanol and stored at –80°C. After completion of the time course, samples were thawed, centrifuged to remove precipitated proteins, and the supernatant was analyzed by HPLC to quantify the release of Rifalogue.

### 4.8 Bacterial Enzyme‐Responsive Drug Release Assay

To evaluate azoreductase‐triggered antibiotic release, a suspension of USA300 was prepared at 1 × 10^5^ CFU/mL. Different AAC constructs were added to a final concentration of 200 nm and incubated at 37°C under shaking. Aliquots were collected at specified time intervals, immediately quenched with methanol, and stored at –80°C. After final collection, samples were centrifuged to remove precipitated proteins, and the supernatant was subjected to HPLC analysis to quantify released Rifalogue.

### 4.9 Preparation of DyLight 680‐Labeled and pH rodo^TM^‐Labeled AAC Conjugates

pH rodo^TM^ Red SE and DyLight 680‐labeled conjugates were synthesized following the manufacturer's instructions. In particular, the conjugates were incubated with the respective dyes in NaHCO_3_ buffer (pH 8.3) for 3 h, and then transferred to PBS buffer to produce the dye‐labeled conjugates. The pHrodo^TM^‐labeled conjugate exhibited an excitation wavelength of 560 nm and an emission wavelength of 585 nm, while the DyLight 680‐labeled conjugate had an excitation wavelength of 692 nm and an emission wavelength of 712 nm.

### 4.10 Cell Culture and Differentiation

The murine macrophage cell line Raw 264.7 and human cell lines U937, A549, HL‐60, and HUVEC were obtained from pre‐existing laboratory frozen stocks. HBMEC was purchased from the Chinese Academy of Sciences. All cells were maintained at 37°C under 5% CO_2_. Raw 264.7, U937, A549, and HL‐60 cells were cultured in DMEM or RPMI‐1640 medium (Gibco, Cat. No. C12571500BT) supplemented with 10% fetal bovine serum (FBS; Gibco, Cat. No. 10099141C), 100 U/mL penicillin, and 100 µg mL^−^^1^ streptomycin (Gibco, Cat. No. 15140122). HUVEC and HBMEC were cultured in endothelial cell medium (ECM; Gibco, Cat. No. 1001) containing 5% FBS, 100 U/mL endothelial cell growth supplement (ECGS; Gibco, Cat. No. 1052), and 100 U/mL penicillin/streptomycin (Gibco, Cat. No. 0503).

### 4.11 Macrophage Differentiation from Monocytic Cells

To differentiate U937 cells into macrophages, cells in logarithmic growth phase were centrifuged and resuspended in complete RPMI‐1640 medium. Phorbol 12‐myristate 13‐acetate (PMA) was added to a final concentration of 100 ng/mL. Cells were adjusted to a density of 5 × 10^5^ cells/mL and seeded into six‐well plates at 2 mL per well. After 24 h, cells transitioned from suspension to adherent growth, exhibiting morphological changes including increased cell volume, cytoplasmic loosening, nuclear enlargement, and membrane protrusions. Successful differentiation was confirmed by microscopy, and a significant increase in CD68 expression was detected via flow cytometry.

### 4.12 Neutrophil Differentiation from HL‐60 Cells

To induce neutrophil differentiation, HL‐60 cells in logarithmic growth phase were centrifuged and resuspended in complete RPMI‐1640 medium. Dimethyl sulfoxide (DMSO) was added to a final concentration of 1%. Cells were adjusted to a density of 3 × 10^5^ cells/mL and seeded into six‐well plates at 2 mL per well. After 24 h, cells exhibited a transition from suspension to adherent growth, with increasing cell volume and the appearance of granulocyte‐specific nuclear morphologies (kidney‐shaped, lobulated, and bean‐shaped nuclei). Successful differentiation was confirmed by microscopic observation and a marked increase in CD11b expression via flow cytometry.

### 4.13 Confocal Microscopy Analysis of MRSA Internalization Pathway

To visualize the endocytic trafficking of USA300 labeled with green fluorescent protein GFP in Raw 264.7 macrophages, cells were seeded in 8‐chamber plates at 1 × 10^5^ cells per well and infected at an MOI of 10–20.1 h post‐infection, extracellular bacteria were eradicated by treatment with gentamicin (100 µg mL^−^^1^) for 1 h at 37°C. After three washes with PBS, cells were maintained in serum‐free, antibiotic‐free DMEM containing 10% FBS and 50 µg mL^−^^1^ gentamicin (at the MIC level), followed by the addition of AAC. The cells were incubated for 16–18 h at 37°C, washed again with PBS, and successively stained with Magic Red reagent (4 h) and acridine orange (30 min). Imaging was performed using a ZEISS LSM 880/900 laser scanning confocal microscope.

### 4.14 MIC Determination Against Extracellular Bacteria

The MIC against planktonic MRSA (USA300) was assessed using the broth microdilution method. Serial two‐fold dilutions of the antibiotic were prepared in Brain Heart Infusion (BHI) broth in quadruplicate within a 96‐well plate. An exponentially growing bacterial culture was diluted to 1 × 10^4^ CFU/mL and added to each well. After incubation at 37°C with shaking for 18–24 h, bacterial growth was quantified by measuring the optical density at 595 nm. The MIC was defined as the lowest antibiotic concentration that inhibited ≥90% of bacterial growth.

### 4.15 Determination of Intracellular MIC

The intracellular MIC was evaluated against MRSA internalized within Raw 264.7 macrophages. Macrophages were seeded at a density of 1 × 10^5^ cells per well and infected with MRSA at a multiplicity of infection (MOI) of 10–20. To eliminate extracellular bacteria, the culture medium was supplemented with gentamicin (100 µg mL^−^^1^) 1 h post‐infection. After 1 h, the medium was replaced with DMEM supplemented with 10% FBS and 50 µg mL^−^^1^ gentamicin, and the test drug was added. After 24 h of incubation, macrophages were lysed using Hank's balanced salt solution containing 0.1% bovine serum albumin (BSA) and 0.1% Triton X‐100. Serial dilutions of the lysate were prepared in PBS with 0.05% Tween‐20, and viable intracellular bacteria were quantified by plating on brain–heart infusion agar plates.

### 4.16 Both Intra‐ and Extracellular Infection Model

An in vitro intracellular and extracellular infection model was established to evaluate antibacterial efficacy against both intra‐ and extracellular MRSA. Raw 264.7 macrophages were seeded at 1 × 10^5^ cells per mL and serum‐starved for 1 h using DMEM basal medium to enhance phagocytic activity. Cells were then infected with MRSA at a multiplicity of infection (MOI) of 10–20. After 1 h, fetal bovine serum was added to a final concentration of 2% to support cell viability, followed by the addition of gradient concentrations of the test compounds. Bacterial survival (intracellular and extracellular) was assessed 24 h post‐treatment. Macrophages were lysed with Hank's balanced salt solution containing 0.1% bovine serum albumin (BSA) and 0.1% Triton X‐100. Serial dilutions of the lysates and supernatant were plated on brain–heart infusion agar, and viable bacteria were quantified by colony counting.

### 4.17 In Vivo Targeting and Imaging of Bacterial Infection

A muscle infection model was employed to evaluate the in vivo targeting capability of AZO‐AAC. Murine (*n* = 3) were shaved and injected intramuscularly with PBS (20 µL) into the left thigh muscle (uninfected control) and MRSA (5 × 10^7^ CFU/mouse in 20 µL) into the right thigh muscle (infected site). Subsequently, DyLight 680‐labeled AZO‐AAC was administered intravenously. Optical in vivo imaging was performed at predetermined time points (0, 3, 6, 9, 12, and 24 h) using an IVIS imaging system with excitation/emission wavelengths of 680/702 nm. After 24 h, murine were euthanized, and major organs (heart, liver, spleen, lungs, kidneys) along with both thigh muscles were excised for *ex vivo* imaging using IVIS.

### 4.18 In Vivo Therapeutic Efficacy in an Intraperitoneal Infection Model

A peritonitis infection model was established to evaluate the in vivo efficacy of AZO‐AAC. Murine (*n* = 5) were infected intraperitoneally with 1 × 10^8^ CFU of bioluminescent USA300‐*luc* suspended in 100 µL. Successful infection was confirmed via IVIS imaging at 0, 12, and 24 h post‐infection. Subsequently, different therapeutic agents were administered intravenously. Optical in vivo imaging was performed at 36 and 48 h using an IVIS imaging system to monitor bacterial load and treatment response.

### 4.19 Septicemia Infection Model for Evaluating AAC Efficacy

A systemic septicemia model was established by intravenous injection of MRSA (USA300) via the tail vein. Murine (*n* = 4) were infected with 5 × 10^6^ CFU diluted in PBS. By 24 h post‐infection, circulating bacteria are largely cleared by innate immune cells (e.g., neutrophils and NK cells), resulting in a predominantly intracellular infection. Therapeutic interventions were initiated 24 h post‐infection. Vancomycin was administered intravenously at 110 mg kg^−^^1^ daily throughout the study. Experimental treatments (AAC, BD37, and free Rifalogue) were diluted in PBS and delivered as a single intravenous injection at the same time point. All Murine were euthanized on day 4 post‐infection. Kidneys were harvested and homogenized in Trizol using a freeze milling instrument (Shanghai Jingxin). Perform a serial dilution of the homogenate in phosphate‐buffered saline (PBS) containing 0.05% Tween‐20, followed by plating onto BHI agar plates. Hearts and kidneys were collected for histopathological evaluation via H&E staining.

### 4.20 Antimicrobial Activity in a Murine Peritonitis Model

The in vivo efficacy of AAC was evaluated in a murine model of MRSA (USA300) peritonitis. Murine were infected intraperitoneally with 1 × 10^7^ or 2 × 10^7^ CFU of MRSA in 100 µL and, after 24 h, randomly divided into six treatment groups (*n* = 6). Each group received a single intraperitoneal dose of one of the following: PBS, vancomycin, BD37, and free Rifalogue, AZO‐AAC, VA‐AAC, or NCL‐AAC (all at 60 mg kg^−^^1^ based on antibody content). After 24 h of treatment, Murine were injected intraperitoneally with 2 mL of cold HBSS and then euthanized.

Peritoneal fluid was collected to quantify bacterial loads. One‐third was used directly for total CFU enumeration. Another third was centrifuged, and the supernatant was plated to assess extracellular CFUs. The remaining third was treated with lysostaphin (15 µg mL^−^^1^) to eliminate extracellular bacteria, followed by lysis with HBSS containing 0.1% BSA and 0.1% Triton X‐100 to release and quantify intracellular MRSA. Peritoneal lavage fluid was serially diluted in PBS containing 0.05% Tween‐20 and spread onto BHI agar plates. Major organs (liver, heart, and kidneys) were harvested for histopathological assessment using H&E staining.

### 4.21 Statistical Analysis

Data were presented as mean ± SD of three independent experiments. Statistical comparisons between two groups were performed using a two‐tailed unpaired Student's *t*‐test, while comparisons among multiple groups were assessed by one‐way ANOVA followed by Bonferroni's post hoc test. Statistical significance was defined as *p* < 0.05. All graphs and statistical analyses were generated using GraphPad Prism 8.0 (GraphPad Software, Inc., La Jolla, CA, USA).

## Author Contributions

L.L., H.D., J.F., W.Z., and X.Z. conceived and designed this study. Y.L., C.L., and L.L. conducted most experiments and data analysis. F.X., J.W., X.Q., S.H., X.L., and X.K performed parts of the experiments. X.Z., H.D., J.F., and D.X. supervised the study and interpreted results. L.L., Q.C., X.Z., and D.X. wrote and revised the manuscript.

## Conflicts of Interest

The authors declare no conflicts of interest.

## Supporting information




**Supporting File**: advs75541‐sup‐0001‐SuppMat.docx.

## Data Availability

The data that support the findings of this study are available from the corresponding author upon reasonable request.
