# Supplementary material for: Pathogen‐Centric Activation of an Azoreductase‐Responsive Antibody‐Antibiotic Conjugate for the Targeted Eradication of MRSA
Source: Adv Sci (Weinh). 2026 May 11;13(42):e75541. doi: 10.1002/advs.75541 (PMC13336114; doi:10.1002/advs.75541)
Supplement: Supplementary file 1 — Supporting File: advs75541‐sup‐0001‐SuppMat.docx. [file ADVS-13-e75541-s001.docx]

**Supporting Information for**

**Pathogen-centric activation of an azoreductase-responsive antibody-antibiotic conjugate for the targeted eradication of MRSA**

Qi Cheng ^a,b,c,‡^; Lianqi Liu ^b,c,‡^; Chenghua Liu ^b,‡^; Fei Xie ^b,c^; Jingwen Dong ^b,c^; Xiaoyu Qin ^b,c^; Shunxiang Huang ^b,c,d^; Xian Li ^b,c,d^; Xingyuan Kou ^b,c,d^; Hongbin Deng ^e,^*; Jiannan Feng ^b,^*; Wu Zhong ^b,c,^*; Dian Xiao ^b,c,^*; Xinbo Zhou ^b,c,^*

**This file includes:**

Figure S1. Confocal microscopy was used to analyze the lysosomal environment of intracellular bacterial model.

Figure S2. Structure of the Linker-drug compounds (L-1/2/3) and structure of the conjugates (AZO-AAC, VA-AAC, NCL-AAC).

Figure S3. Comparative efficacy chart of both intra- and extracellular infection models on agar Plates.

Figure S4. Antibacterial activity of AZO-AAC in a peritoneal model.

Figure S5. Antibacterial activity of AZO-AAC in a peritoneal model.

Figure S6. H&E scoring were assigned to various murine tissues.

1.Synthetic Procedures

2. Conjugated scheme

3. Characterization Figure


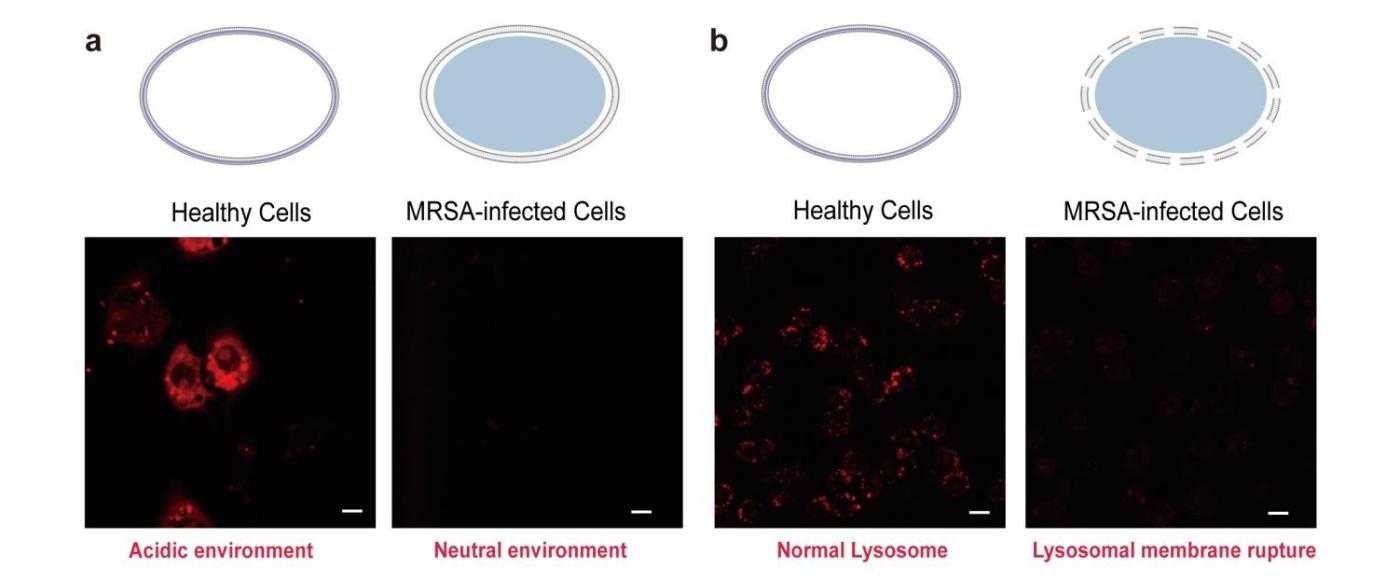


# Figure. S1 Confocal microscopy was used to analyze the lysosomal environment of intracellular bacterial model. **a** The acidity of lysosomes was measured using pH rodo™ reagent. Scale bar = 10 μm. **b** The acidity and alkalinity of lysosomes, as well as the integrity of lysosomal membranes, were measured using the acridine orange reagent. Scale bar = 10 μm.

**
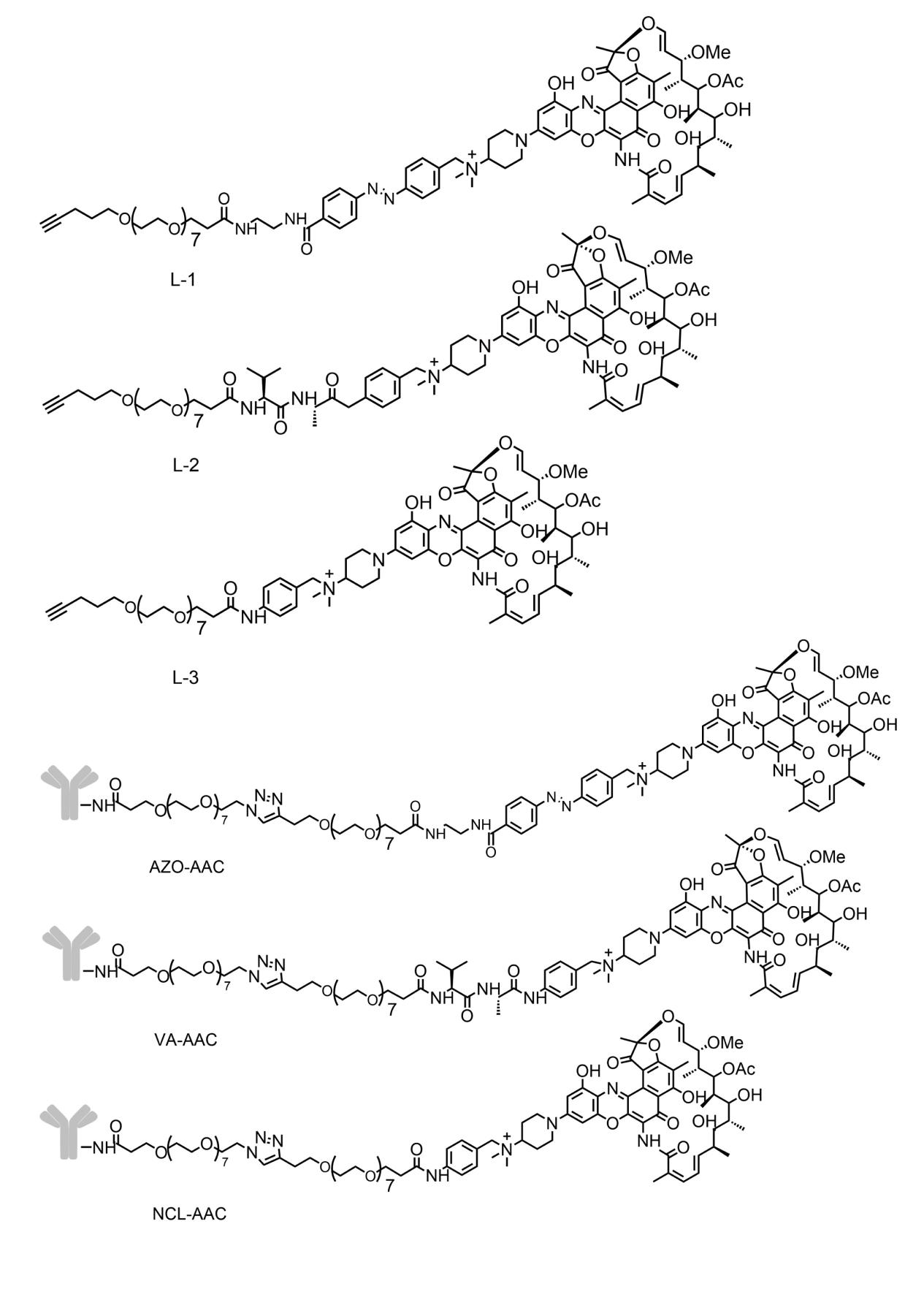
**

Figure. S2 Structure of the Linker-drug compounds (L-1/2/3) and structure of the conjugates (AZO-AAC, VA-AAC, NCL-AAC).


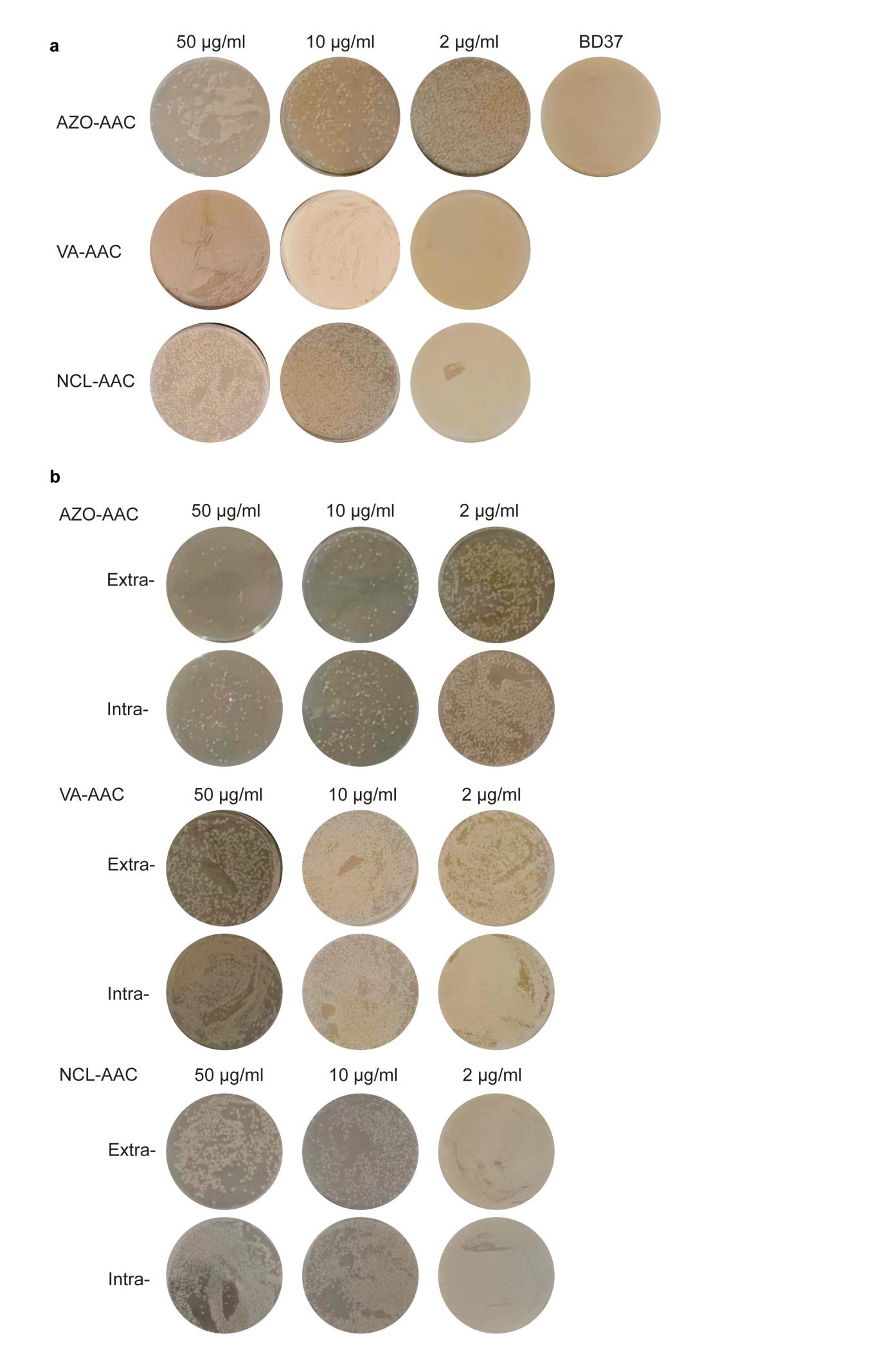


# Figure. S3 Comparative efficacy chart of both intra- and extracellular infection models on agar Plates. **a** Representative CFUs photos of infected cell at different dose under different treatments. **b** Representative CFUs in total, intra- and extracellular fractions photos of infected cell under different treatments.

**
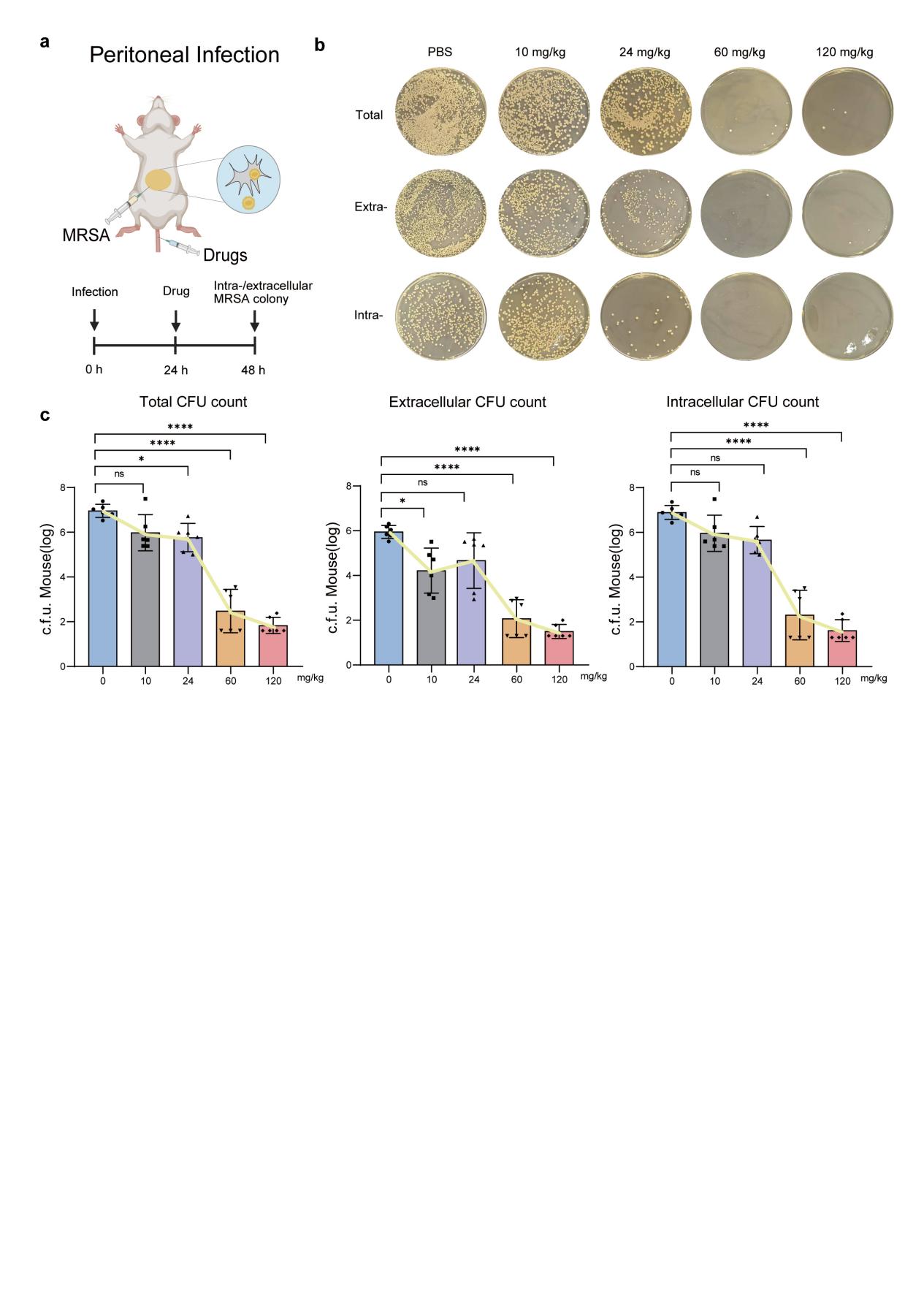
**

# Figure. S4 Antibacterial activity of AZO-AAC in a peritoneal model. **a** Schematic of the peritonitis animal model: Each mouse received an intravenous injection of 1×10^7 CFUs of MRSA. An both intra- and extracellular infection model was established 24 hours post-infection. The peritoneal fluid was collected 24 hours after drug administration, and intra- and extracellular bacteria were isolated for monoclonal counting. **b** Representative CFUs in total, intra- and extracellular fractions photos of infected cell under different treatments. **c** Total, intra- and extracellular fractions CFUs were determined 24 h after the different treatments, n = 6. Data were analyzed by Ordinary one-way ANOVA (Multiple comparisons). *p<0.05, **p<0.005, ***p<0.0002, ****p<0.0001, ns, not significant. Figure was created with BioRender.com, with permission.

**
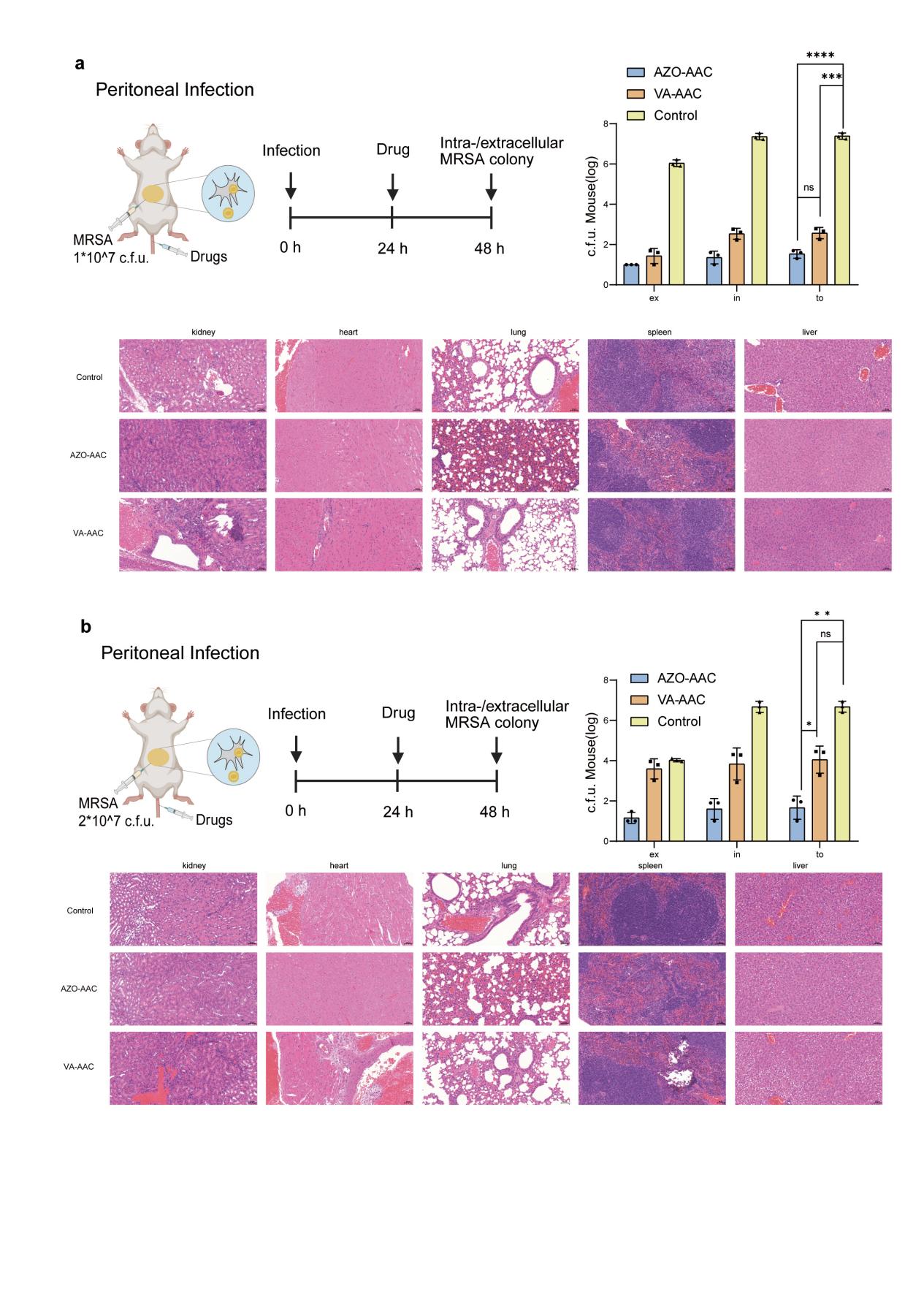
**

Figure. S5 Antibacterial activity of AZO-AAC in a peritoneal model. Schematic of the peritonitis model: Each mouse received an intravenous injection of different CFUs load of MRSA. An both intra- and extracellular infection model was established 24 hours post-infection. The peritoneal fluid was collected 24 hours after drug administration, and intra- and extracellular bacteria were isolated for monoclonal counting.Total, intra- and extracellular fractions CFUs were determined 24 h after the different treatments, n = 3. And Representative sections of heart, kidney, lung, spleen and liver from different treatments stained with H&E. Data were analyzed by Ordinary one-way ANOVA (Multiple comparisons). *p<0.05, **p<0.005, ***p<0.0002, ****p<0.0001, ns, not significant. **a** 1×10^7 c.f.u MRSA; the administered dose 60mg kg⁻¹. **b** 2×10^7 c.f.u MRSA; the administered dose 60mg kg⁻¹. Figure was created with BioRender.com, with permission.


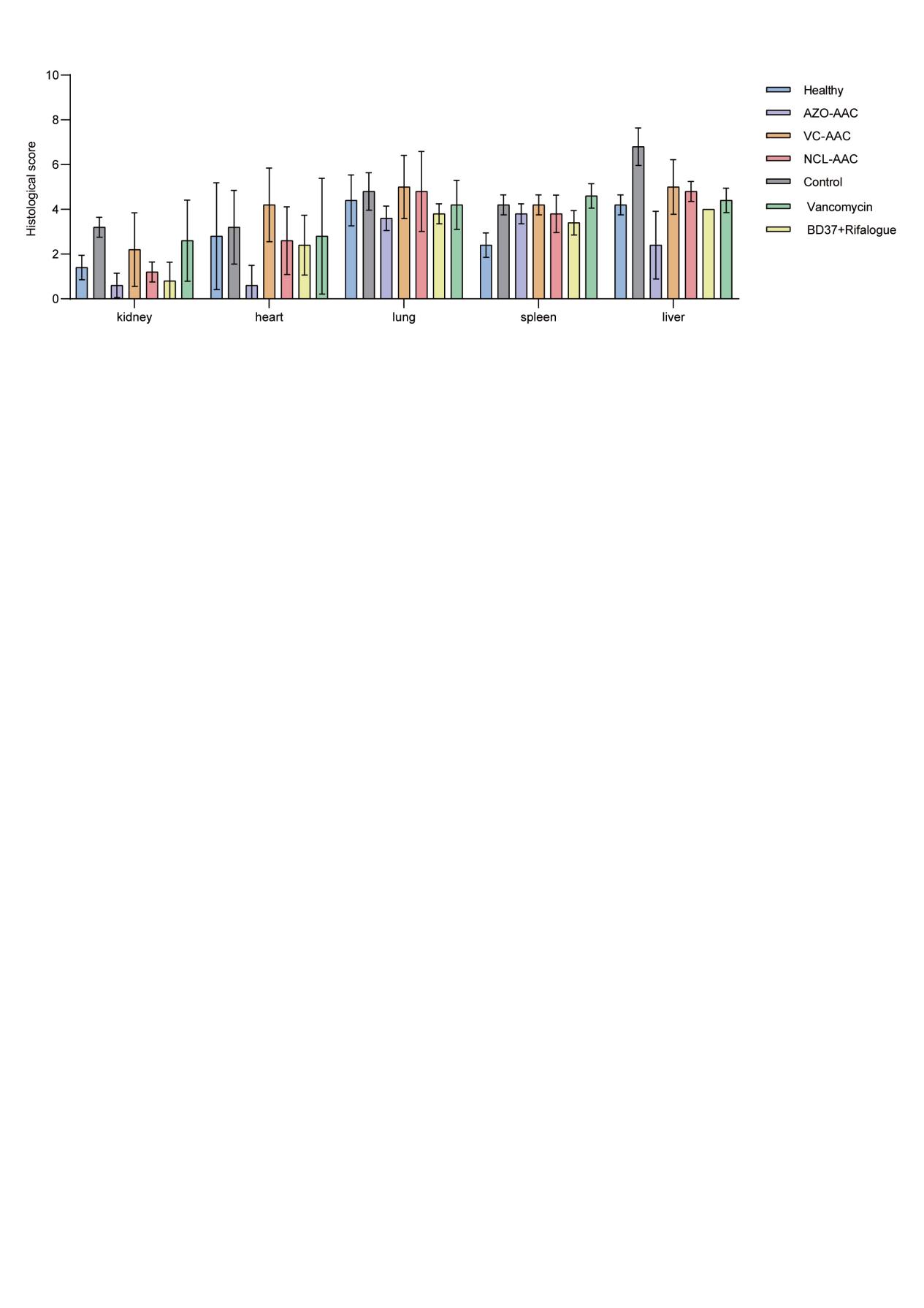


Figure. S6 H&E scoring were assigned to various murine tissues.

1. **Synthetic Procedures**

*4-[(1E)-[4-(hydroxymethyl)phenyl]diazenyl]-N-(29-oxo-30-aza-5,8,11,14,17,20,23,26-octaoxadotriacont-1-yn-32-yl)benzamide* **(M2)**

Compound propargyl-PEG8-acid (0.15 g, 0.344 mmol) NHS(0.036mg, 0.316mmol) DIC (0.054mg, 0.431mmol)was dissolved in DCM (2 mL), and then stir at room temperature overnight.After at least 24 hours, compound M1 (0.086 g, 0.287 mmol) and DIPEA (0.044 mg, 0.344 mmol) dissolved in 200 μl DMF were added to the reaction solution. After completion of the reaction, the solvent was concentrated under reduced pressure.The residue obtained was redissolved with EA and then pure water was added to achieve separation of the organic and aqueous layers. The EA layer was evaporated to obtain compound M2 as an orange solid (0.232 g, 90% yield). ESI-MS m/z (M+H)^+^ calculated for C_37_H_55_N_4_O_11_ 731.4, found 731.4.

*4-[(1E)-[4-(chloromethyl)phenyl]diazenyl]-N-(29-oxo-30-aza-5,8,11,14,17,20,23,26-octaoxadotriacont-1-yn-32-yl)benzamide* **(M3)**

Compound M2(0.103 g, 0.144 mmol) was dissolved in DCM (2 mL), and Dichlorosulfoxide (0.026 mg, 0.216 mmol) was then added in three batches at 8°C with stirring.The mixture was stirred at 8°C for 4 h. After completion of the reaction, the solvent was concentrated under reduced pressure.The obtained residue was redissolved with EA, and then PE was slowly added dropwise to precipitate insoluble matter. The insoluble solid was separated by filtration, and the filter cake was washed with ether to obtain compound M3 as a orange solid (0.09 g, 95% yield). ESI-MS m/z M calculated for C_37_H_54_ClN_4_O_10_ 748.4, found 748.4.

*{1-[(4E,6E,8S,9S,10R,11R,12R,13S,14R,15S,16Z,19S)-13-acetoxy-9,11,26,35-tetrahydroxy-15-methoxy-4,8,10,12,14,19,36-heptamethyl-3,20,33-trioxo-2,24-diaza-18,31-dioxahexacyclo[17.13.6.022,34.021,37.023,32.025,30]octatriaconta-1(32),4,6,16,21(22),23(24),25(26),27,29,34(35),36-undecaen-28-yl]hexahydropyridin-4-yl}({4-[(1E)-[4-(29,34-dioxo-30,33-diaza-5,8,11,14,17,20,23,26-octaoxatetratriacont-1-yn-34-yl)phenyl]diazenyl]phenyl}methyl)dimethylammonium* **(L-1)**

Compound M3 (50 mg, 0.068 mmol) and Rifalogue were dissolved in DMF (400 μl), then TBAI (12 mg, 0.03 mmol) and DIPEA (19 mg, 0.30 mmol) were dissolved in DMF (40 μl) were added with 400 μl DMF. The mixture was Oscillated at 40°C for 6 days. After completion of the reaction, the solvent was concentrated under reduced pressure. Further purification was performed by silica column chromatography to give compound L-1 as a light blue-black solid (38 mg, 78% yield).^1^H-NMR (600 MHz, CDCl_3_): δ 0.95-1.07 (s, 9H), 1.27 (d, 3H), 1.51 (s, 3H), 1.91 (s, 1H) , 1.93(m, 5H), 1.94 (s, 1H), 2.01(m, 5H), 2.03-2.15 (s, 4H), 2.45-2.60 (m, 4H), 2.79 (s, 1H), 2.83 (m, 6H), 2.88 (s, 1H), 3.05 (s, 1H), 3.11-3.47 (m, 13H), 3.58-3.87 (m, 32H), 3.93 (s, 1H), 4.04-4.21 (m, 2H), 4.37-4.50 (s, 3H), 5.21-5.32 (s, 2H), 6.16-6.38 (m, 4H), 6.52 (s, 1H), 7.05 (m, 5H). ESI-MS m/z M^+^ calculated for C_88_H_113_N_8_NaO_23_^+^ 1626.8, found 1626.8.

The synthetic routes of compounds L-2 and L-3 followed the previous work (*Adv Sci 2025, 12 (31), e02270*). Compounds L-2 and L-3 were determined by MS (L-2: ESI-MS m/z M^+^ calculated for C_85_H_118_N_7_O_24_^+^ 1621.8, found 1621.8; L-3: ESI-MS m/z M^+^ calculated for C_77_H_104_N_5_O_22_⁺1450.7, found 1450.7).

1. **Conjugated scheme**

Anti-*S. aureus* antibody BD37 in HEPES buffer (20 mM, pH=7) was treated with NHS-PEG8-Azide (~6 equivalents relative to the whole antibody) for 12 h，the reaction to obtain the intermediate. To the intermediate were added the desired compounds L-1 (20 equivalents) in dimethylacetamide (DMA), subsequently joined copper sulfate pentahydrate and THPTA were premixed for 5 min, then Na·VC was added and mixed for 10 min, for 24 h. The conjugates were exchanged into HEPES buffer (pH = 7) using Sephadex G-25S to remove the free L-1 to give the AZO-AAC and stored at –80 °C before use for analysis and testing. The VA-AAC and NCL-AAC were were prepared according to the same method.

1. **Characterization Figure**


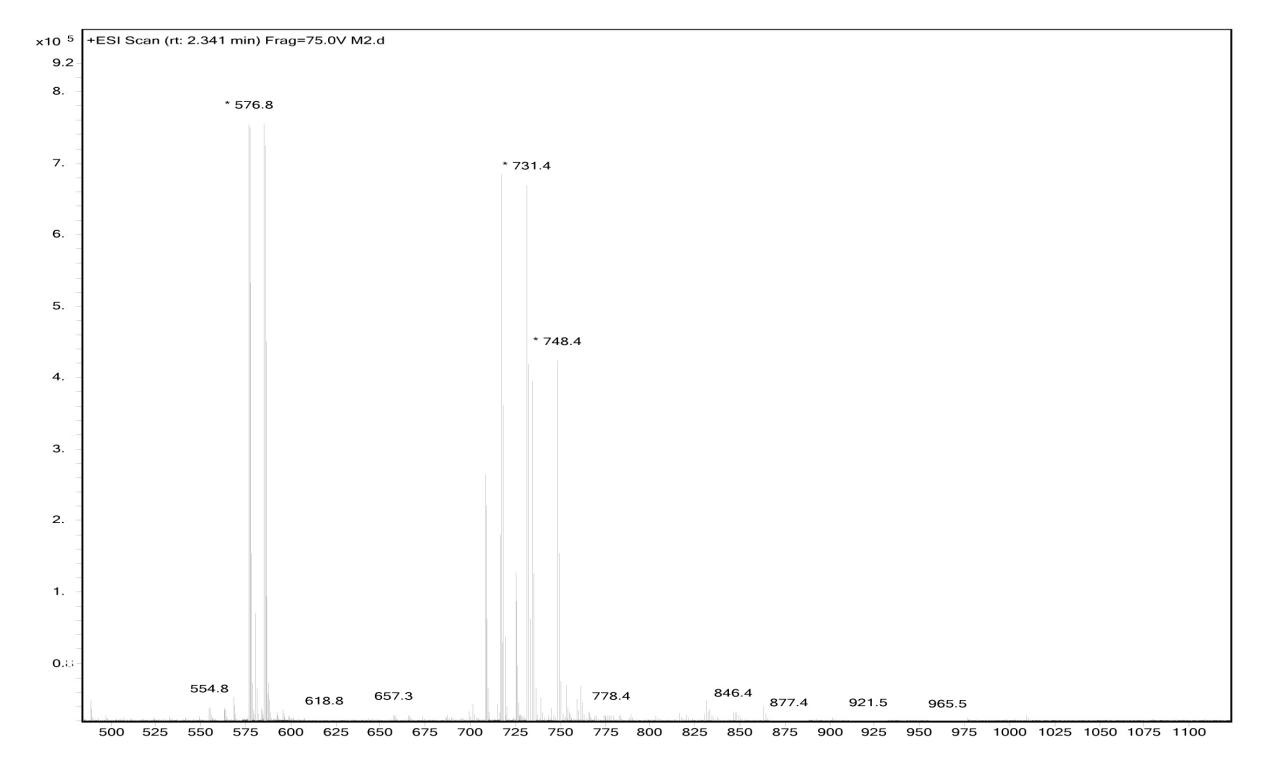


The MS spectrum of M2.


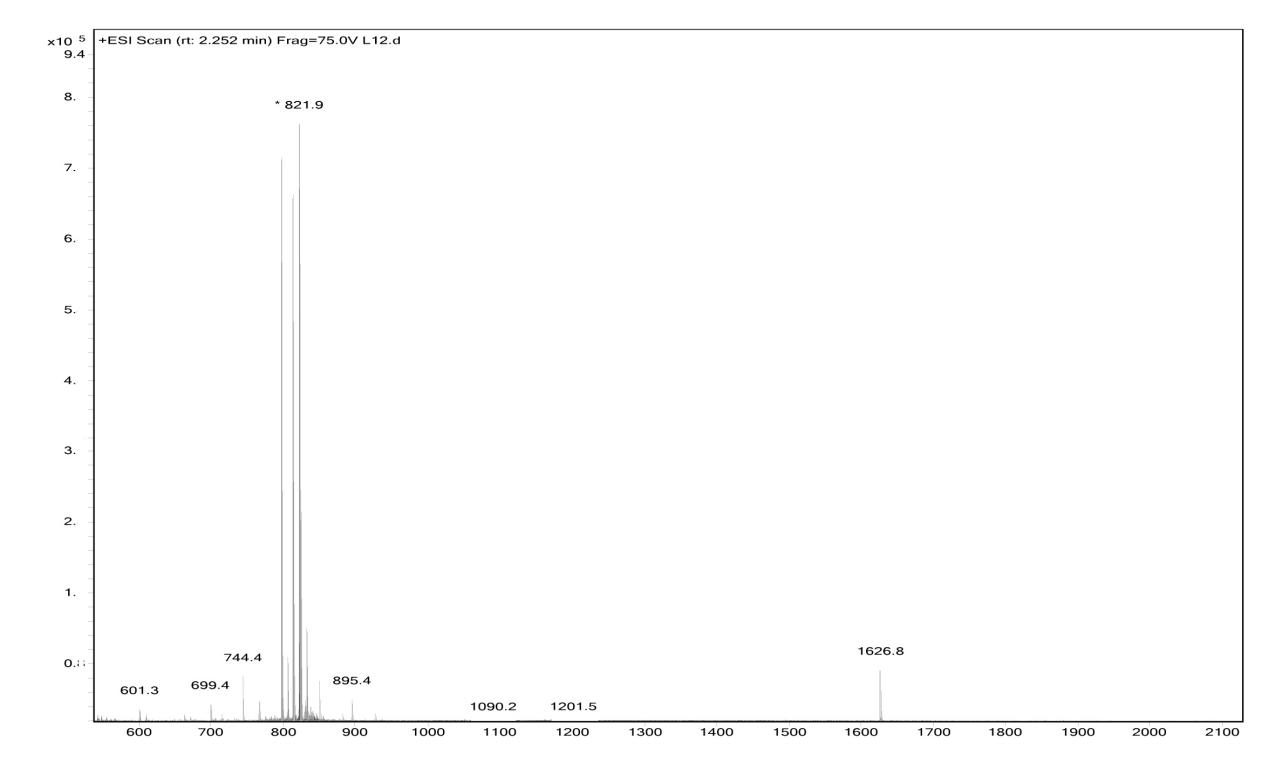


The MS spectrum of L-1.


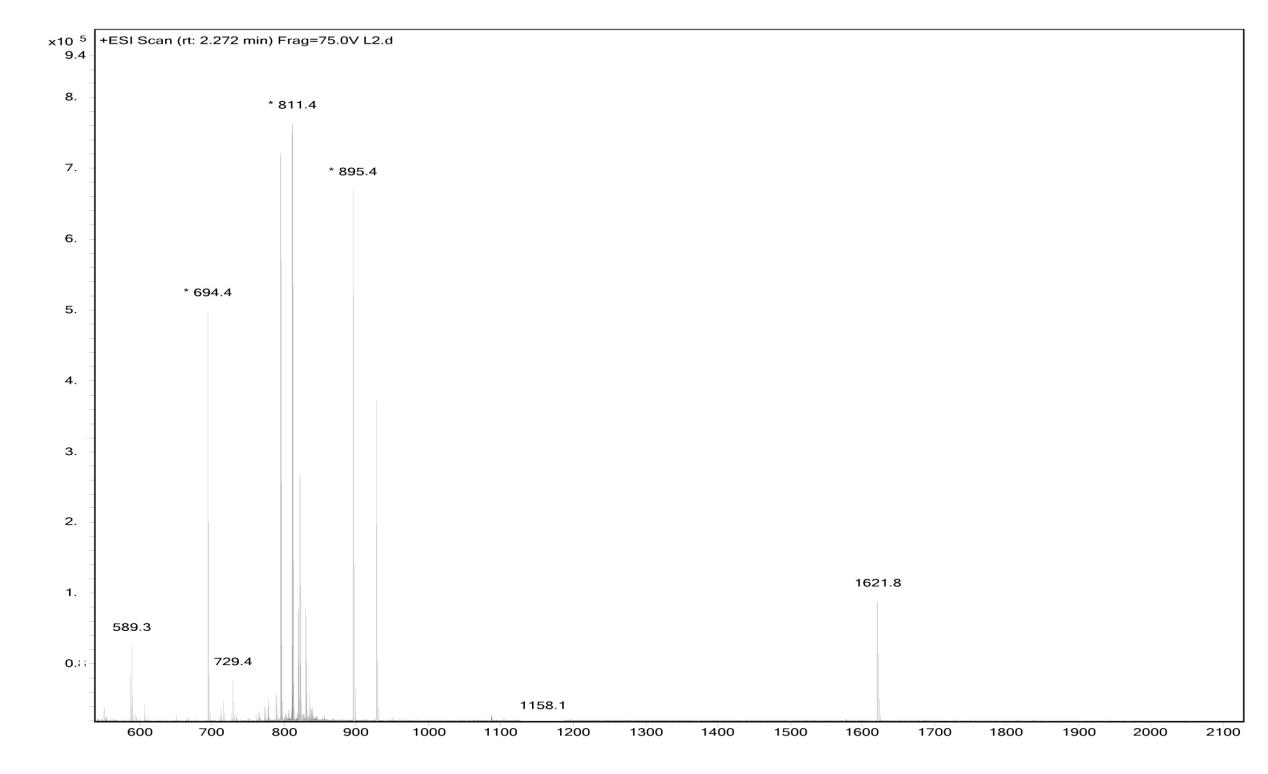


The MS spectrum of L-2.


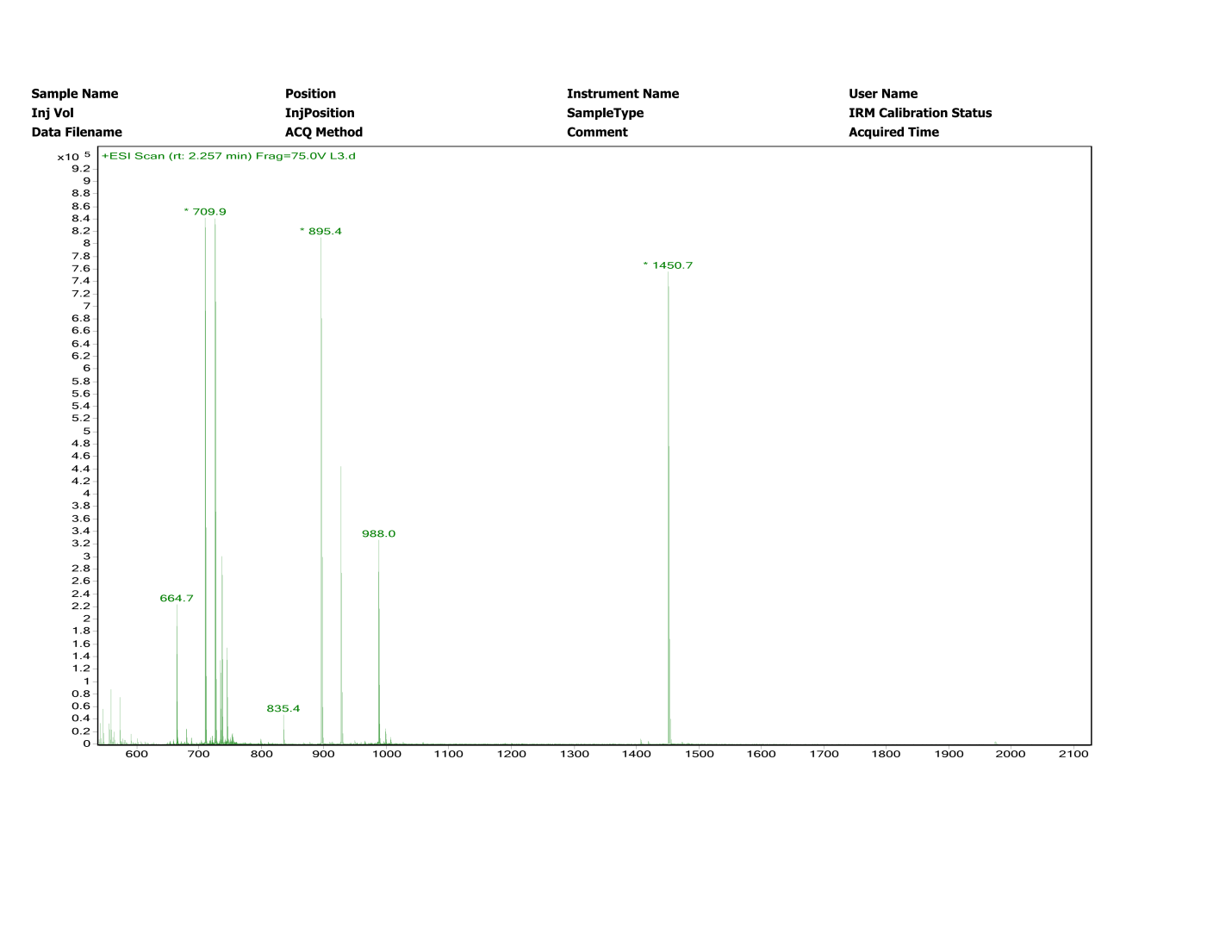


The MS spectrum of L-3.


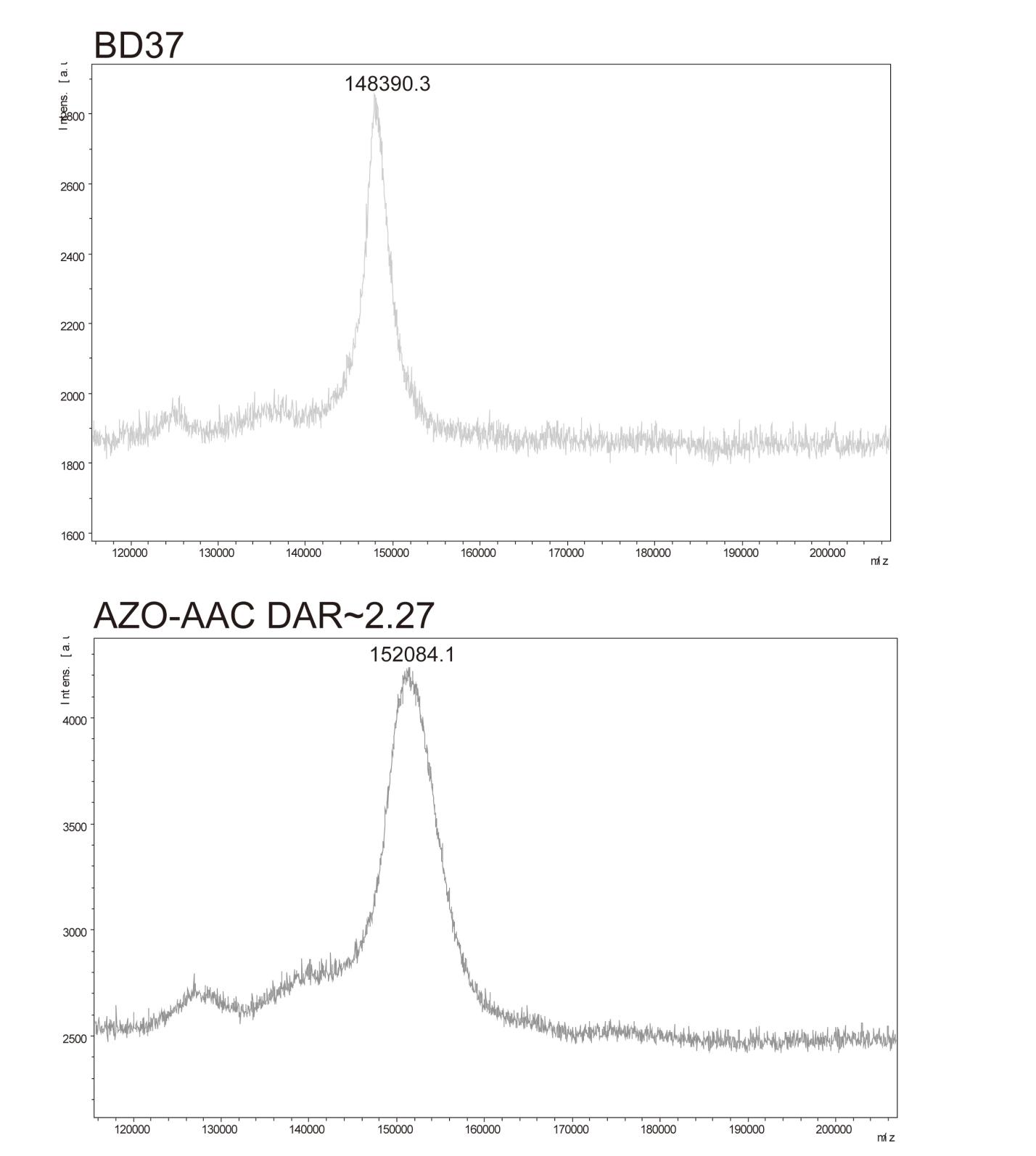
The The MALDI-TOF analysis of BD37 and AZO-AAC.


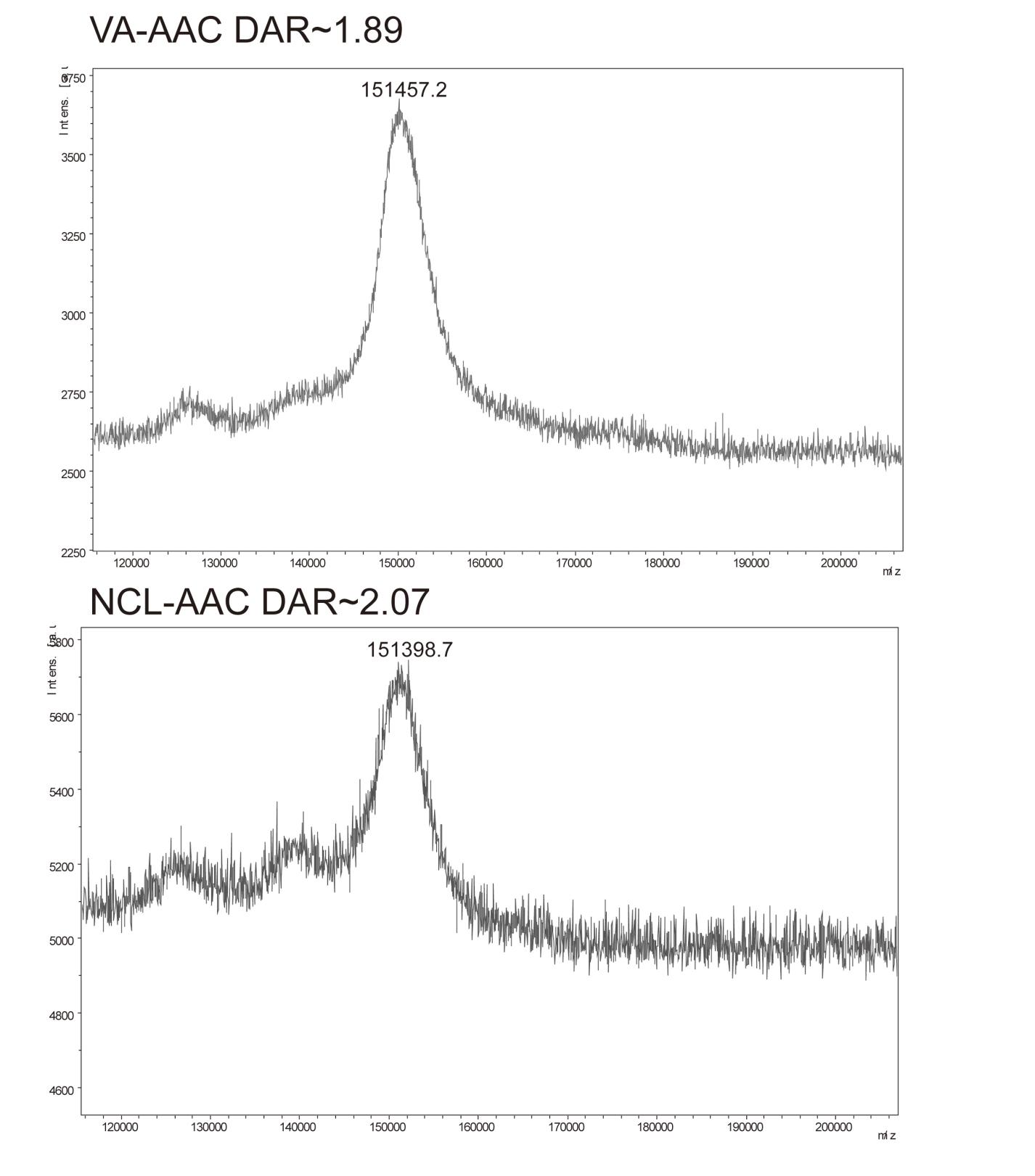


The MALDI-TOF analysis of VA-AAC and NCL-AAC.
